# Entangled states shaping with CV states of definite parity

**DOI:** 10.1038/s41598-022-05336-2

**Published:** 2022-01-28

**Authors:** Dmitry A. Kuts, Sergey A. Podoshvedov

**Affiliations:** Laboratory of Quantum Information Processing and Quantum Computing, South Ural State University (SUSU), Lenin Av. 76, Chelyabinsk, Russia

**Keywords:** Optics and photonics, Physics, Quantum physics, Qubits, Single photons and quantum effects

## Abstract

We present a new method to entangle continuous variable (CV) states of certain parity and photonic states for the purpose of generating optical hybrid cluster (HC) states. To do it we introduce two families of the CV states of definite parity which stems from single mode squeezed vacuum (SMSV) state. Potential to apply the CV states of certain parity is high. We report on the generation of the even/odd Schrödinger cat state like (SCS-like) states whose fidelities with even/odd SCS of amplitude of $4.2$ are more of $0.99$, when 30,31 photons are detected in auxiliary mode of input SMSV state initially mixed with single photon. We show that the quantum efficiency of a photon number resolving (PNR) detector is crucial to maintaining the success rate of even/odd SCSs generator at an acceptable level. The scheme with delocalized photon implements deterministic imperfect entanglement operation between macro and micro states. We show that the beam splitter implements the two-qubits operation $control-Z$ (CZ) for input CV states of definite parity and photonic states, provided that certain result is detected in measurement mode. An extension of the entangling operation for two entangled delocalized photons (TEDP) allows one to realize three-qubit HC state. Seven-qubit HC state is the result of conjunction of two three-qubit HC states through TEDP state.

## Introduction

The concept of entanglement is a fundamental property in quantum mechanics that underlies photonic quantum computer^1–3^. Optical photonic states are easy to transform into each other, photons propagate in a medium with maximum speed. Quantum states of photons are free of cooling systems due to their extremely weak interaction with the environment. Nevertheless, photons do not interact with each other directly which affect the implementation of quantum optical protocols properly^4^. Optical methods of shaping of optical entanglement have been traditionally implemented based on either continues variables or discrete variables of quantum light. An entanglement of cluster states has attracted much attention as a universal resource for one-way quantum computation^5–7^. A serious progress has been done towards creating optical hybrid entangled states^8–12^ and exploring new capabilities in quantum information processing^13–15^. Delivering the entanglement in a scalable manner is highly desirable for development of optical quantum technologies. As the measurement-induced approach is more practical, there have been many demonstrations of method^16–20^ for measurement-based state engineering, involving those applicable to SCSs generation^21–26^. Advancement in detector technology capable to number resolution^27,28^ allows one to move to a qualitatively new stage of state engineering schemes^29–32^.

Here, we propose model of interaction between CV states of certain parity with photonic states with its extension on the case of generation of more complex hybrid entangled states of light. To use the model in practice, we introduce two families of CV states of certain parity. The CV states of definite parity are generated by subtraction of a number of photons from original SMSV state. If even SMSV state only passes through the beam splitter followed by the detection of $n$ photons in an auxiliary mode, then the measured-induced state remains even (i.e., containing exclusively even Fock states) in the case even measurement outcome and changes its parity to the opposite odd one in the case of odd measurement outcome. On the contrary, if we superimpose original SMSV state of with a single photon, the parities of the heralded CV states are reversed in comparison with the case of using vacuum in the auxiliary mode. The potential of the new CV states can range from generator of even/odd SCSs, entangled operations to create hybrid cluster states, to quantum metrology. If the number of photons detected in auxiliary mode is large enough, the conditional CV state in its properties can approach to SCS-like states^21,29^, including the cases when they approximate target even/odd SCSs of amplitude $4.2$ with fidelity more of $0.99$. If SMSV state is mixed with DF with the subsequent registration of the measuring outcome in an auxiliary mode, then the operation leads to the generation of macro–micro entanglement, regardless of the outcome and parameters of optical scheme. The number of cases of when the scheme acts as perfect entangling operation and synthesizes maximum hybrid entanglement is quite large. In many cases, applications require multipartite entanglement including larger number of qubits^33,34^. We use the entanglement mechanism to create three-qubit and seven-qubit HC states. The seven-qubit HC state is realized by connecting two three-qubit HC states by means of an additional TEDP state. In implementation of the HC state, beam splitter can realize two-qubit CZ gate for the CV state of definite parity and photonic states provided that two specific measurement outcomes are observed in auxiliary mode.

## Results

### Families of the CV states

**Figure 1:**
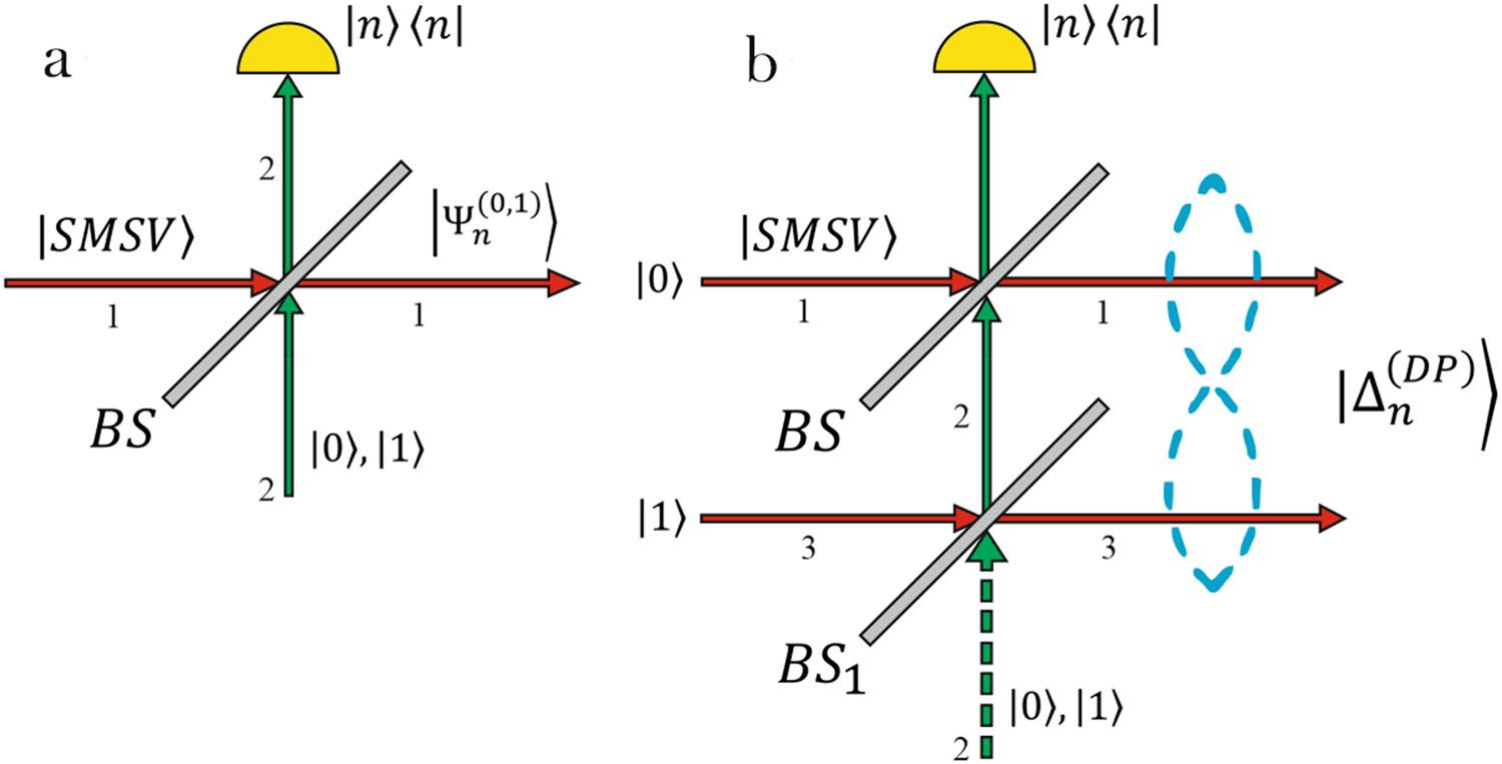
(**a,b**) Optical schemes adjusted for measurement-induced generation of CV states of definite parity (left subfigure) and deterministic implementation of CV-DV entanglement (right subfigure) by superimposing input SMSV state with either vacuum, single photon or delocalized photon created by lower BS in the picture on the right.

To illustrate the basic technique of conditional shaping of hybrid entangled states composed of CV states of definite parity and photonic states, initially, we introduce the families of the CV states of definite parity. The experimental scheme shown in Fig. 1a is used to generate the states. The even SMSV state

1 $$ \left| {SMSV} \right\rangle = \frac{1}{{\sqrt {{\text{cosh}}r} }}\mathop \sum \limits_{n = 0}^{\infty } b_{2n} \left| {2n} \right\rangle = \frac{1}{{\sqrt {{\text{cosh}}r} }}\mathop \sum \limits_{n = 0}^{\infty } \left( \frac{tanhr}{2} \right)^{n} \frac{{\sqrt {\left( {2n} \right)!} }}{n!}\left| {2n} \right\rangle , $$

is sent into a beam splitter (BS)

2 $$ BS_{12} = \left[ {\begin{array}{*{20}c} t & { - \fancyscript{r}} \\ {\fancyscript{r}} & t \\ \end{array} } \right] $$

which is considered to be no longer necessarily balanced^29^, where $t$ and $\fancyscript{r}$ are the real transmittance and reflectance coefficients satisfying the normalization condition $t^{2} +\fancyscript{r}^{2} = 1$ and subscript $\left( {12} \right)$ denote the mixed optical modes. Reflected photons from the BS are monitored with PNR detector. Now, the optical transition edge sensors (TES) has been improved and utilized for the resolution of the number $n$ of photons arriving for detection^27,28^. Extraction of a certain number of photons either $2m$ or $2m + 1$ from the SMSV in an indistinguishable manner with loss of all information from which Fock states of the original superposition the photons are subtracted generates $2m/2m + 1$ heralded states leaving the output state with a well-defined parity either even or odd in dependency on the parity of original states and parity of the measurement outcome.

The detection of even number $n = 2m$ of photons in second auxiliary mode corresponds to the conditional generation of even CV states i.e. ones containing exclusively even Fock states

3 $$ \begin{gathered} \left| {{\Psi }_{2m}^{\left( 0 \right)} } \right\rangle = L_{2m}^{\left( 0 \right)} \mathop \sum \limits_{n = 0}^{\infty } b_{{2\left( {n + m} \right)}} t^{2n} \sqrt {\frac{{\left( {2\left( {n + m} \right)} \right)!}}{{\left( {n + m} \right)!}}} \left| {2n} \right\rangle  \hfill \\ \quad = \frac{1}{{\sqrt {Z^{{\left( {2m} \right)}} } }}\mathop \sum \limits_{n = 0}^{\infty } y^{n} \frac{{\sqrt {\left( {2\left( {n + m} \right)} \right)!} }}{{\left( {n + m} \right)!}}\sqrt {\left( {2n + 1} \right)\left( {2n + 2} \right) \ldots 2\left( {n + m} \right)} \left| {2n} \right\rangle , \hfill \\ \end{gathered} $$

where $L_{2m}^{\left( 0 \right)} = \sqrt {coshr} \left( {t^{2} } \right)^{m} /y^{m} \sqrt {Z^{{\left( {2m} \right)}} }$, $Z^{\left( m \right)} = d^{2m} Z/dy^{2m}$ with $Z \equiv Z\left( y \right) = Z^{\left( 0 \right)} = 1/\sqrt {1 - 4y^{2} }$ and $y = t^{2} tanhr/2$ (the mathematical derivation of the normalization term is presented in the section Methods). The parameter $y$ can take values in the range $0\le y\le 0.5$, provided that $r>0$ the condition we are going to adhere to in the analysis. The condition $y=0$ formally takes place in the case of either $r=0$ or $t=0$ which is not a case for our consideration. The opposite case $y=0.5$ takes place only in the limiting case of $t=1$ and $r\to \infty$ that going beyond the scope of physical consideration. In general, the parameter $y$ can take on rather small values $y\ll 0.5$, especially in experimental setup with a high-refractive beam splitter in combination with a small value of the squeezing parameter $r < 1$. Note that the subscript indicates the number of subtracted photons in the second auxiliary mode while superscript points to the initial auxiliary DV state. The success probability to implement the $2m$ heralded state is equal to

4 $$ P_{2m}^{\left( 0 \right)} = \frac{1}{coshr}\left( {\frac{{1 - t^{2} }}{{t^{2} }}} \right)^{2m} \frac{{y^{2m} }}{{\left( {2m} \right)!}}Z^{{\left( {2m} \right)}} . $$

In particular, in the absence of a click in the measurement mode $\left( {m = 0} \right)$ the following conditional state

5 $$ \begin{gathered} \left| {{\Psi }_{0}^{\left( 0 \right)} } \right\rangle = \frac{1}{\sqrt Z }\mathop \sum \limits_{n = 0}^{\infty } y^{n} \frac{{\sqrt {\left( {2n} \right)!} }}{n!}\left| {2n} \right\rangle\hfill \\ \quad = \left( {1 - t^{4} tanh^{2} r} \right)^{1/4} \mathop \sum \limits_{n = 0}^{\infty } y^{n} \frac{{\sqrt {\left( {2n} \right)!} }}{n!}\left| {2n} \right\rangle \hfill \\ \end{gathered} $$

is generated with success probability $P_{0}^{\left( 0 \right)} = Z/coshr$.

Let us assume that the PNR detector in the measuring mode registers an odd $2m + 1$ number of photons. Then, the $2m + 1$ heralded odd CV state is produced

6 $$ \begin{gathered} \left| {{\Psi }_{2m + 1}^{\left( 0 \right)} } \right\rangle = L_{2m + 1}^{\left( 0 \right)} \mathop \sum \limits_{n = 0}^{\infty } b_{{2\left( {n + m + 1} \right)}} t^{2n} \sqrt {\frac{{\left( {2\left( {m + n + 1} \right)} \right)!}}{{\left( {2n + 1} \right)!}}} \left| {2n + 1} \right\rangle\hfill \\ \quad = \sqrt {\frac{y}{{Z^{{\left( {2m + 1} \right)}} }}} \mathop \sum \limits_{n = 0}^{\infty } y^{n} \frac{{\sqrt {\left( {2\left( {n + m + 1} \right)} \right)!} }}{{\left( {n + m + 1} \right)!}}\sqrt {\left( {2n + 2} \right)\left( {2n + 3} \right) \ldots 2\left( {n + m + 1} \right)} \left| {2n + 1} \right\rangle . \hfill \\ \end{gathered} $$

with the success probability

7 $$ P_{2m + 1}^{\left( 0 \right)} = \frac{1}{coshr}\left( {\frac{{1 - t^{2} }}{{t^{2} }}} \right)^{2m + 1} \frac{{y^{2m + 1} }}{{\left( {2m + 1} \right)!}}Z^{{\left( {2m + 1} \right)}} , $$

where $L_{2m + 1}^{\left( 0 \right)} = \sqrt {coshr} \left( {t^{2} } \right)^{m + 1} /\sqrt {y^{2m + 1} Z^{{\left( {2m + 1} \right)}} }$ is the normalization factor. The total success probability $\mathop \sum \limits_{m = 0}^{\infty } \left( {P_{2m}^{\left( 0 \right)} + P_{2m + 1}^{\left( 0 \right)} } \right) = 1$ is normalized.

Consider modification of the initial experiment in Fig. 1a with the inclusion of a single photon in second mode in addition to the SMSV in Eq. (1) in first mode of the BS in Eq. (2). Removing the $n-$ photon Fock state from the original SMSV by detection of $n$ photons in second port of the BS generates odd CV states

8 $$ \begin{gathered} \left| {{\Psi }_{0}^{\left( 1 \right)} } \right\rangle = \frac{1}{{Z^{3/2} }}\mathop \sum \limits_{n = 0}^{\infty } y^{n} \frac{{\sqrt {\left( {2n} \right)!} }}{n!}\sqrt {2n + 1} \left| {2n + 1} \right\rangle\hfill \\ \quad = \left( {1 - t^{4} tanh^{2} r} \right)^{3/4} \mathop \sum \limits_{n = 0}^{\infty } y^{n} \frac{{\sqrt {\left( {2n} \right)!} }}{n!}\sqrt {2n + 1} \left| {2n + 1} \right\rangle , \hfill \\ \end{gathered} $$

in the case of nothing detection $\left( {n = 0} \right)$ in the measurement mode with success probability

9 $$ P_{0}^{\left( 1 \right)} = \frac{{\left( {1 - t^{2} } \right)}}{{L_{0}^{\left( 0 \right)2} }} = \frac{{\left( {1 - t^{2} } \right)}}{coshr}Z^{3} , $$

where $L_{0}^{\left( 1 \right)} = \sqrt {coshr} /Z^{3/2}$ is the normalization factor and

10 $$ \begin{gathered} \left| {{\Psi }_{2m}^{\left( 1 \right)} } \right\rangle = L_{2m}^{\left( 1 \right)2} \mathop \sum \limits_{n = 0}^{\infty } b_{{2\left( {n + m} \right)}} t^{2n} \sqrt {\frac{{\left( {2\left( {n + m} \right)} \right)!}}{{\left( {2n + 1} \right)!}}} \left( {1 - \frac{2n + 1}{{2m}}\frac{{1 - t^{2} }}{{t^{2} }}} \right)\left| {2n + 1} \right\rangle\hfill \\ \quad = \frac{1}{{\sqrt {G_{2m} } }}\mathop \sum \limits_{k = 0}^{\infty } y^{n} \frac{{\sqrt {\left( {2\left( {n + m} \right)} \right)!} }}{{\left( {n + m} \right)!}}\sqrt {\left( {2n + 2} \right)\left( {2n + 3} \right) \ldots 2\left( {n + m} \right)} \left( {1 - \frac{{\left( {2n + 1} \right)}}{2m}\frac{{1 - t^{2} }}{{t^{2} }}} \right)\left| {2n + 1} \right\rangle , \hfill \\ \end{gathered} $$

in the case of detection of even number $n = 2m$ photons with the success probability

11 $$ P_{2m}^{\left( 1 \right)} = \frac{{\left( {2m} \right)^{2} }}{coshr}\frac{{\left( {1 - t^{2} } \right)^{2m - 1} }}{{\left( {t^{2} } \right)^{2m - 2} }}\frac{{y^{2m} }}{{\left( {2m} \right)!}}G_{2m} , $$

where $G_{2m} = Z^{{\left( {2m - 1} \right)}} /y - \left( {1 - t^{2} } \right)Z^{{\left( {2m} \right)}} /\left( {mt^{2} } \right) + \left( {1 - t^{2} } \right)^{2} \left( {yZ^{{\left( {2m} \right)}} } \right)^{\left( 1 \right)} /\left( {2mt^{2} } \right)^{2}$ and $L_{2m}^{\left( 1 \right)} = t^{2m} \sqrt {coshr} /y^{m} \sqrt {G_{2m} }$. Subtraction of odd number $n = 2m + 1$ photons from original SMSV produces even CV superpositions

12 $$ \begin{gathered} \left| {{\Psi }_{2m + 1}^{\left( 1 \right)} } \right\rangle = L_{2m + 1}^{\left( 1 \right)} \mathop \sum \limits_{n = 0}^{\infty } b_{{2\left( {n + m} \right)}} t^{2n} \sqrt {\frac{{\left( {2\left( {n + m} \right)} \right)!}}{{\left( {2n} \right)!}}} \left( {t^{2} - \frac{2n}{{2m + 1}}\frac{{1 - t^{2} }}{{t^{2} }}} \right)\left| {2n} \right\rangle\hfill \\ \quad = \frac{1}{{\sqrt {G_{2m + 1} } }}\mathop \sum \limits_{n = 0}^{\infty } y^{n} \frac{{\sqrt {\left( {2\left( {n + m} \right)} \right)!} }}{{\left( {n + m} \right)!}}\sqrt {\left( {2n + 1} \right)\left( {2n + 2} \right) \ldots 2\left( {n + m} \right)} \left( {1 - \frac{2n}{{2m + 1}}\frac{{1 - t^{2} }}{{t^{2} }}} \right)\left| {2n} \right\rangle , \hfill \\ \end{gathered} $$

with success probability

13 $$ P_{2m + 1}^{\left( 1 \right)} = \frac{{\left( {2m + 1} \right)^{2} }}{coshr}\frac{{\left( {1 - t^{2} } \right)^{2m} }}{{\left( {t^{2} } \right)^{2m - 1} }}\frac{{y^{2m} }}{{\left( {2m + 1} \right)!}}G_{2m + 1} , $$

where $G_{2m + 1} = Z^{{\left( {2m} \right)}} - 2\left( {1 - t^{2} } \right)yZ^{{\left( {2m + 1} \right)}} /\left( {\left( {2m + 1} \right)t^{2} } \right) + \left( {1 - t^{2} } \right)^{2} y\left( {yZ^{{\left( {2m + 1} \right)}} } \right)^{\left( 1 \right)} /\left( {\left( {2m + 1} \right)t^{2} } \right)^{2}$ and $L_{2m + 1}^{\left( 1 \right)} = t^{2m} \sqrt {coshr} /y^{m} \sqrt {G_{2m + 1} }$. Direct calculations allow one to verify performance of the normalization condition $\mathop \sum \limits_{m = 0}^{\infty } \left( {P_{2m}^{\left( 1 \right)} + P_{2m + 1}^{\left( 1 \right)} } \right) = 1$.

In general, the introduced states of the certain parity are similar to each other in their form. This is especially true for even and odd CV states in Eqs. (3,6) as well as the states in Eqs. (8,10,12). Nevertheless, difference in the form of the CV states exists, in particular, expressed by an additional factor in brackets in the states in Eqs. (10,12) unlike the CV states in Eqs. (3,6). In particular, the states in Eqs. (3,6) depend on only one parameter $y$ determined by the parameters $\left( {t,r} \right)$ of the optical scheme, while the states in Eqs. (8,10,12) depend on two parameters: $y$ and additionally $t$. This makes it possible to divide the introduced states into two families of states: ${\Psi }^{\left( 0 \right)}$ and ${\Psi }^{\left( 1 \right)}$ family, each of which can also be divided into two subfamilies: even $2m -$heralded ${\Psi }^{\left( 0 \right)}$, odd $2m + 1 -$heralded ${\Psi }^{\left( 0 \right)}$, odd $2m -$heralded ${\Psi }^{\left( 1 \right)}$ and even $2m + 1 -$heralded ${\Psi }^{\left( 1 \right)}$ states. Each of the subfamilies contains an infinite number of states that can be used for optical quantum information processing. In general, the families of the ${\Psi }^{\left( 0 \right)}$ and ${\Psi }^{\left( 1 \right)}$ states originate from the SMSV and their forms are completely determined by the set two experimental parameters $\left( {t,r} \right)$.

### Transition ${{\varvec{\Psi}}}^{\left( 0 \right)} ,{{\varvec{\Psi}}}^{\left( 1 \right)}$ states from squeezed to SCS states

**Figure 2:**
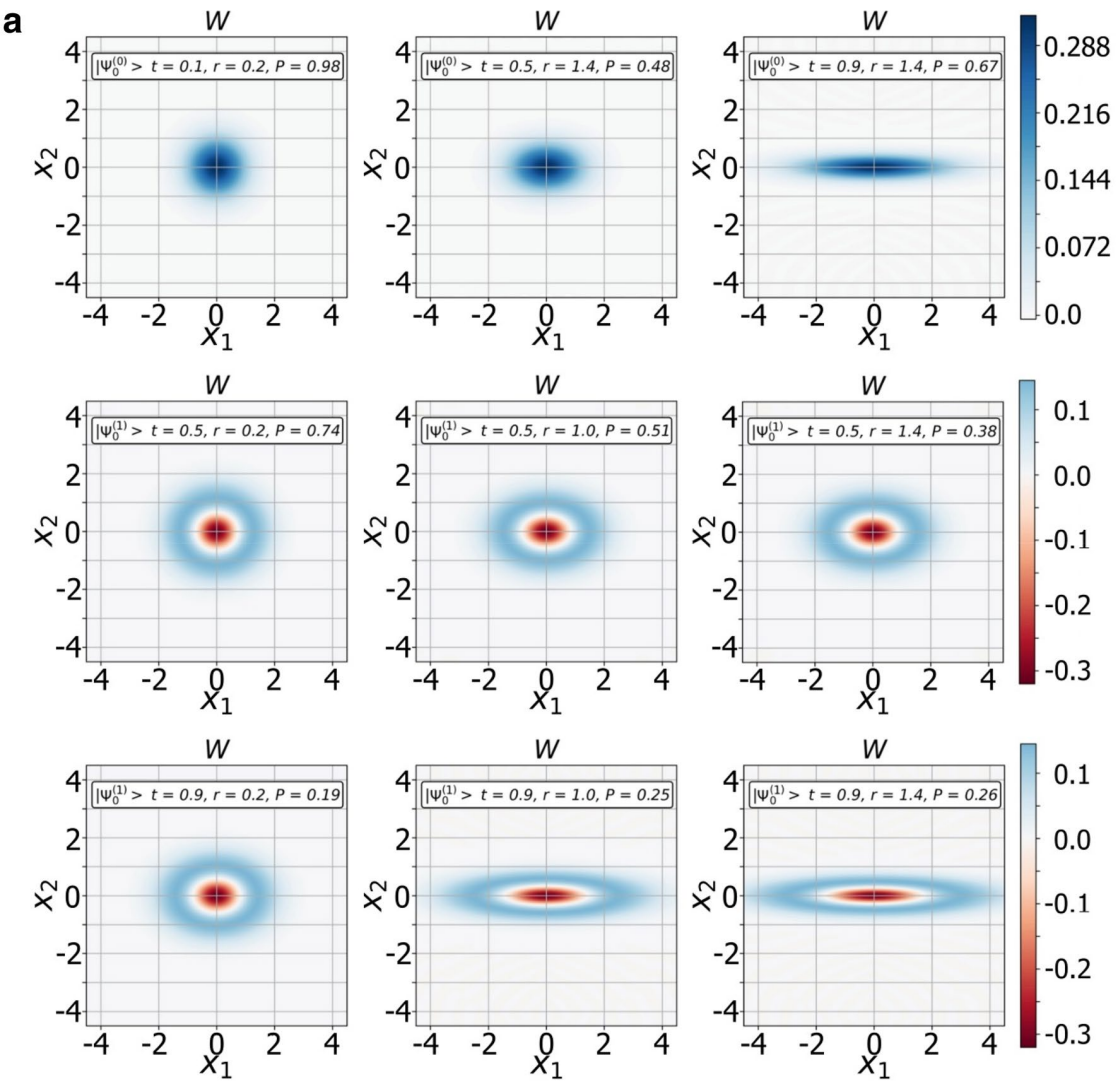

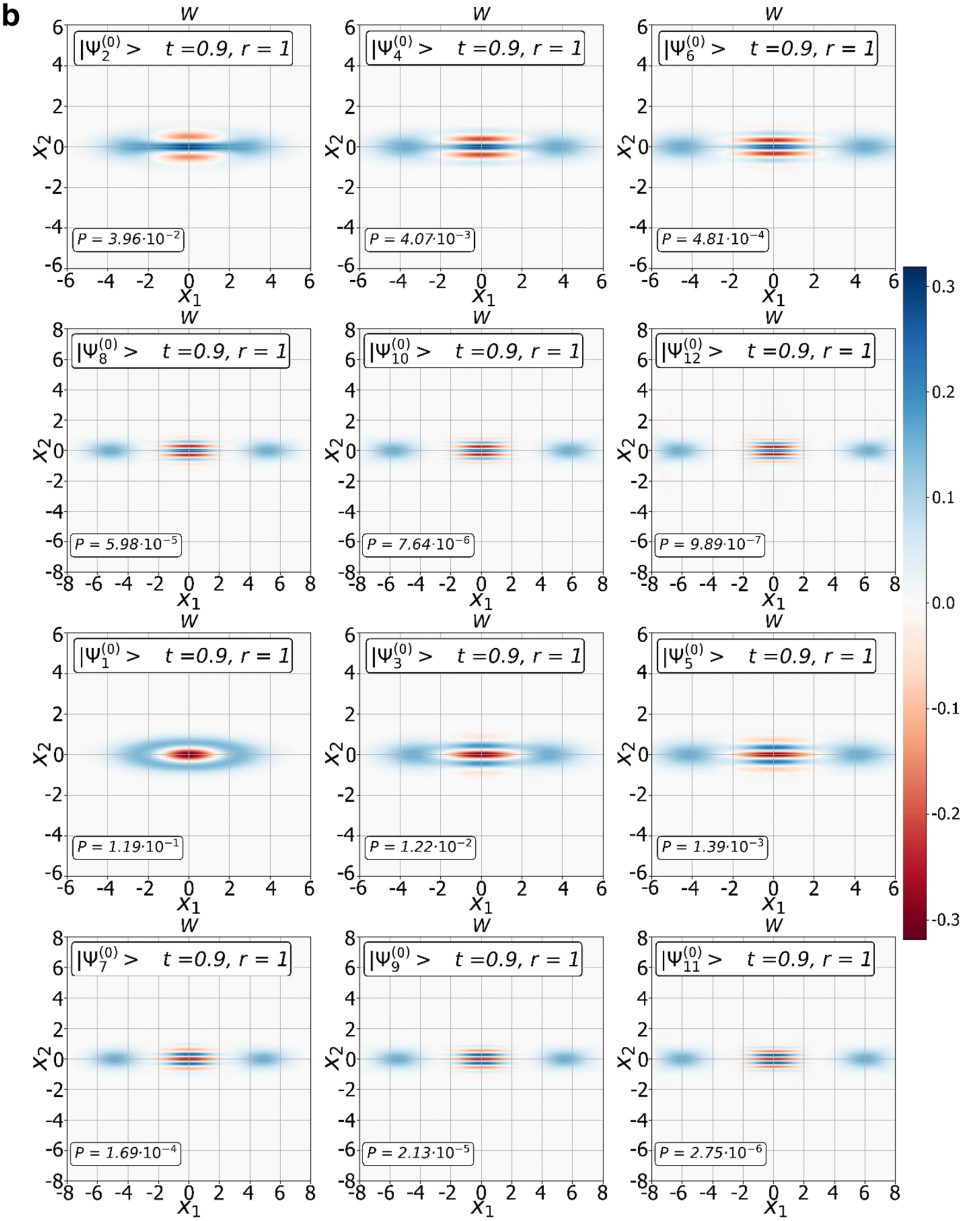

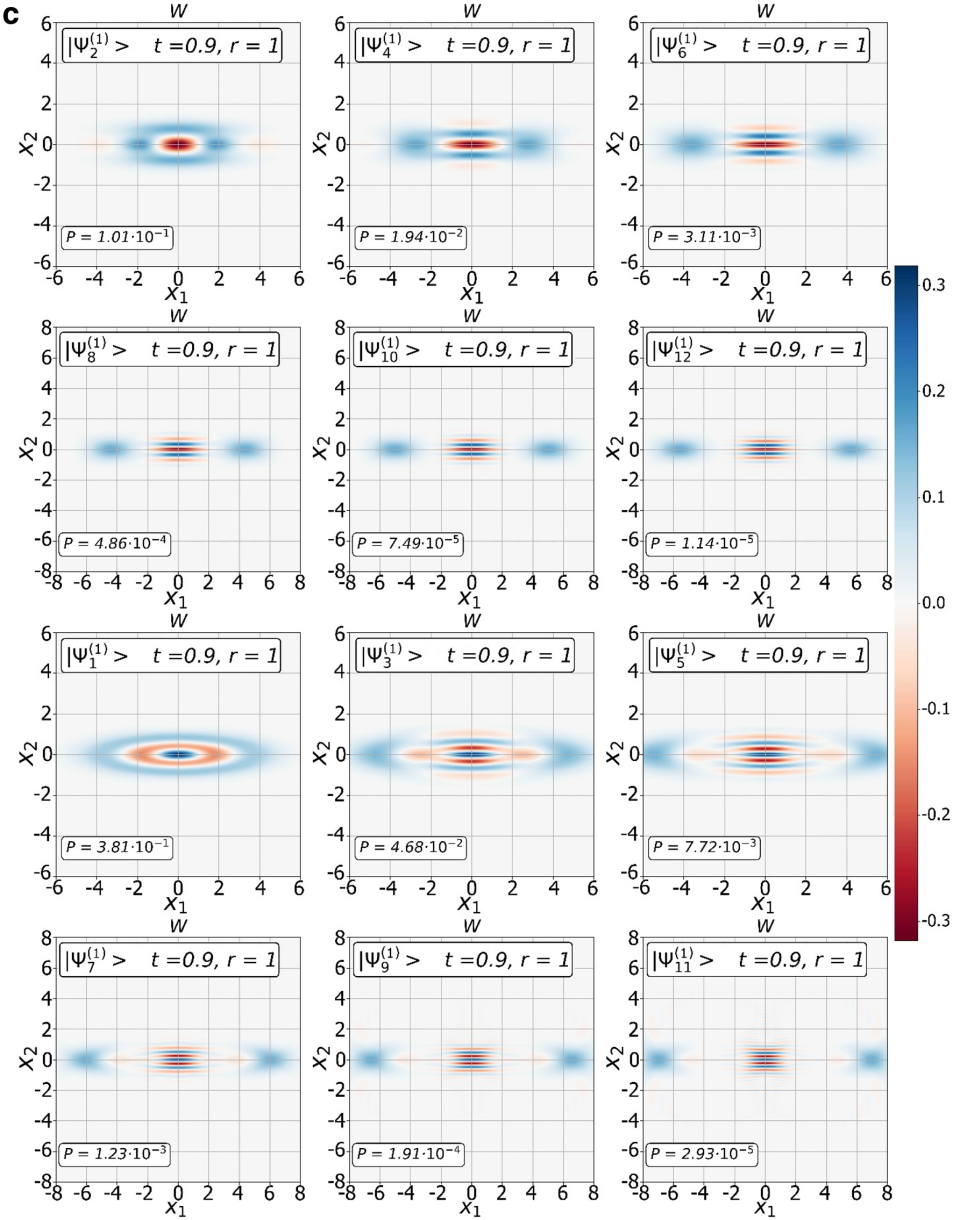
Transition from squeezed to SCS-like states with increase of number of photon subtracted. (**a**) Plots of the Wigner functions $W$ of the conditional states $|{\Psi }_{0}^{\left(0\right)}\rangle$ (top three graphs) and $|{\Psi }_{0}^{\left(1\right)}\rangle$ (next six plots) as functions of the quadrature components ${X}_{1}$ and ${X}_{2}$ for different values of $t$ and $r$. (**b**) Plots of the Wigner functions $W$ of the states from ${\Psi }^{\left(0\right)}$ family: $|{\Psi }_{2m}^{\left(0\right)}\rangle$ (top six plots) and $|{\Psi }_{2m+1}^{\left(0\right)}\rangle$ (next six plots) as functions of the quadrature components ${X}_{1}$ and ${X}_{2}$ for the same values of $t=0.9$ and $r=1$ but with different values of $m$ changing by one when moving from one plot to another. (**c**) Plots of the Wigner functions $W$ of the states from ${\Psi }^{\left(1\right)}$ family: $|{\Psi }_{2m}^{\left(1\right)}\rangle$ (top six plots) and $|{\Psi }_{2m+1}^{\left(1\right)}\rangle$ (next six plots) as functions of the quadrature components ${X}_{1}$ and ${X}_{2}$ for the same values of $t=0.9$ and $r=1$ but with different values of $m$ changing by one when moving from one plot to another.

**Figure 3:**
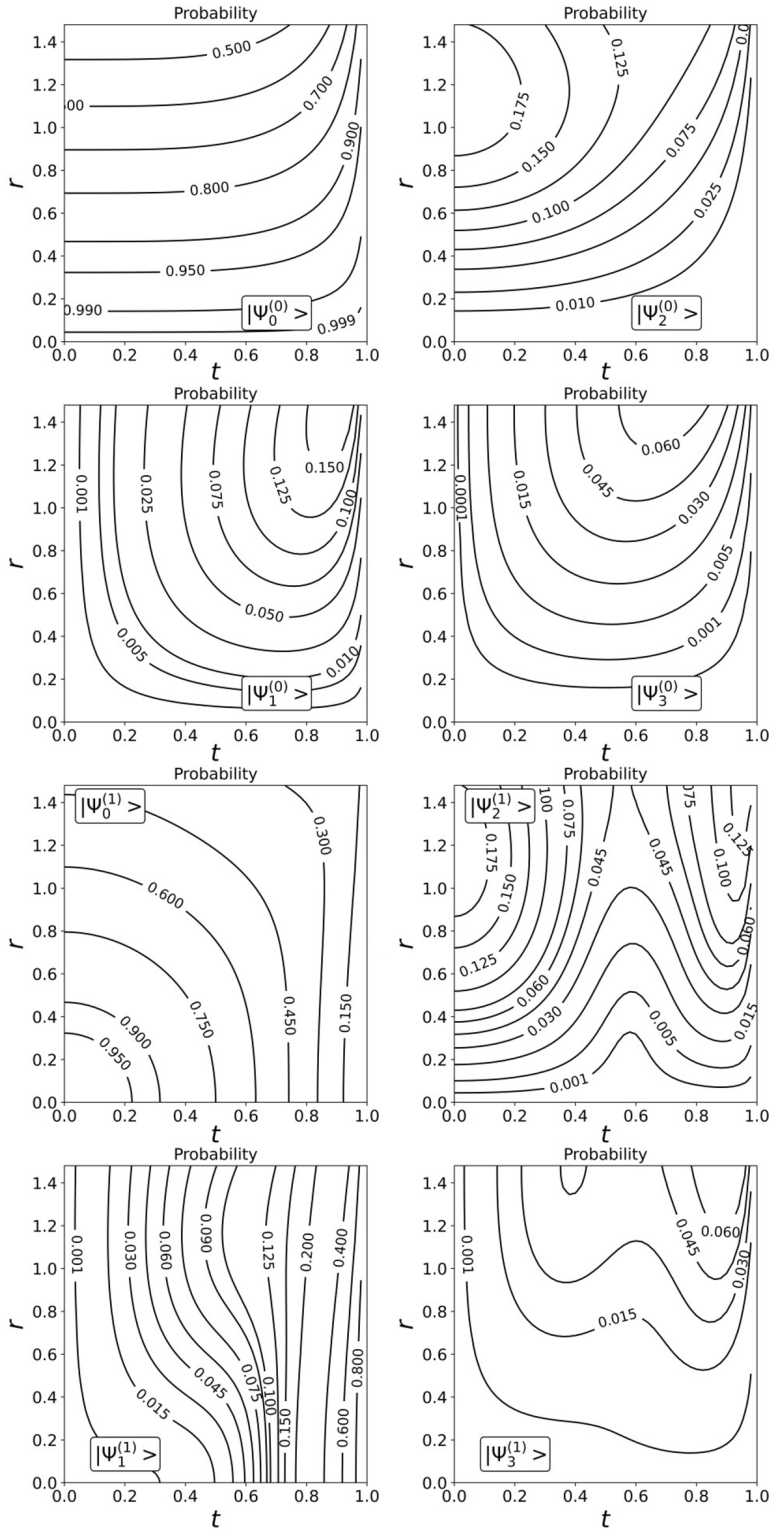
Contour lines for some levels of the success probability $\left(P=const\right)$ as a function of $t$ and $r$ for measurement-induced states with different $m$.

We have found a way to shape ${\Psi }^{\left( 0 \right)}$ and ${\Psi }^{\left( 1 \right)}$ families of the CV nonclassical states of certain parity. Since the ${\Psi }^{\left( 0 \right)}$ and ${\Psi }^{\left( 1 \right)}$ CV states stem from the original SMSV with reduced noise in one of the quadrature components, they may also have similar properties. Indeed, modeling of Wigner functions of the states confirms it. In Fig. 2a, we show the dependence of the Wigner functions $W$ of the measurement-induced states $\left| {{\Psi }_{0}^{\left( 0 \right)} } \right\rangle$ and $\left| {{\Psi }_{0}^{\left( 1 \right)} } \right\rangle$ on the quadrature components $X_{1}$ and $X_{2}$ for different values $\left( {t,r,m = 0} \right)$. The squeezing of the quadrature amplitude $X_{2}$ depends on the beam splitter parameter $t$ in addition to the squeezing parameter $r$ of the initial SMSV state, moreover, contribution of the parameter $t$ can be significant. An increase in the number of photons extracted from the SMSV leads to a qualitative change in behavior of the $2m,2m + 1 -$ heralded states. Figures 2b, c show Wigner's functions of the CV states of definite parity from ${\Psi }^{\left( 0 \right)}$ and ${\Psi }^{\left( 1 \right)}$ families with different values of the parameter $m$ for $t = 0.9$ and $r = 1$. The Wigner functions of the conditioned states exhibit distinct stand-alone peaks along the quadrature component $X_{1}$ and interference behavior along the quadrature component $X_{2}$. The change in the interference pattern occurs in accordance with the behavior of even/odd SCSs when positive values of the Wigner function near the center $\left({X}_{1}=0,{X}_{2}=0\right)$ for an even SCS are replaced by negative values for an odd SCS. The transition from squeezed to SCS-like states is more brightly arisen with increasing the number of the Fock state detected in auxiliary mode. We also show in Fig. 3 contour outlines $\left(P=const\right)$ of the success probabilities ${P}_{2m,2m+1}^{\left(0\right)}$ in Eqs. (4,7) and ${P}_{2m,2m+1}^{\left(0\right)}$ in Eqs. (9,11,3) with $m$ varying from $0$ up to $1$ in dependence on $t$ and $r$. As can be seen, there are ranges of parameter values $\left(t,r\right)$ at which the success probability can take large enough values. The graph becomes useful when choosing the values of the parameters $\left(t,r\right)$ to estimate success probability for real experiment.

**Figure 4:**
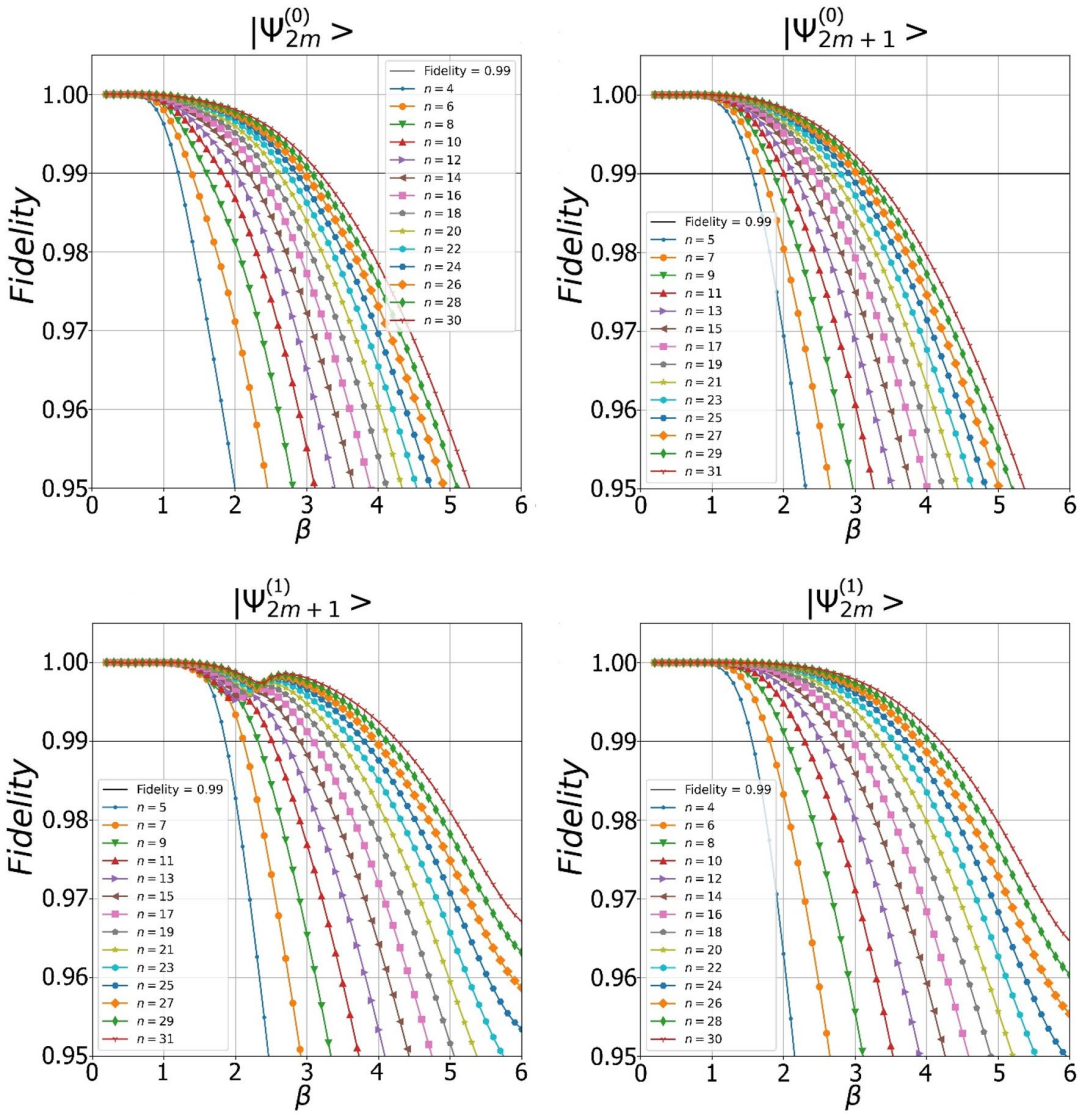
Dependencies of fidelities between the even/odd states from ${\Psi }^{\left(0\right)}$ and ${\Psi }^{\left(1\right)}$ families and real even/odd SCSs on its amplitude $\beta$. The more photons are extracted from the original SMSV state, the higher the fidelity of the output state is observed.

**Figure 5:**
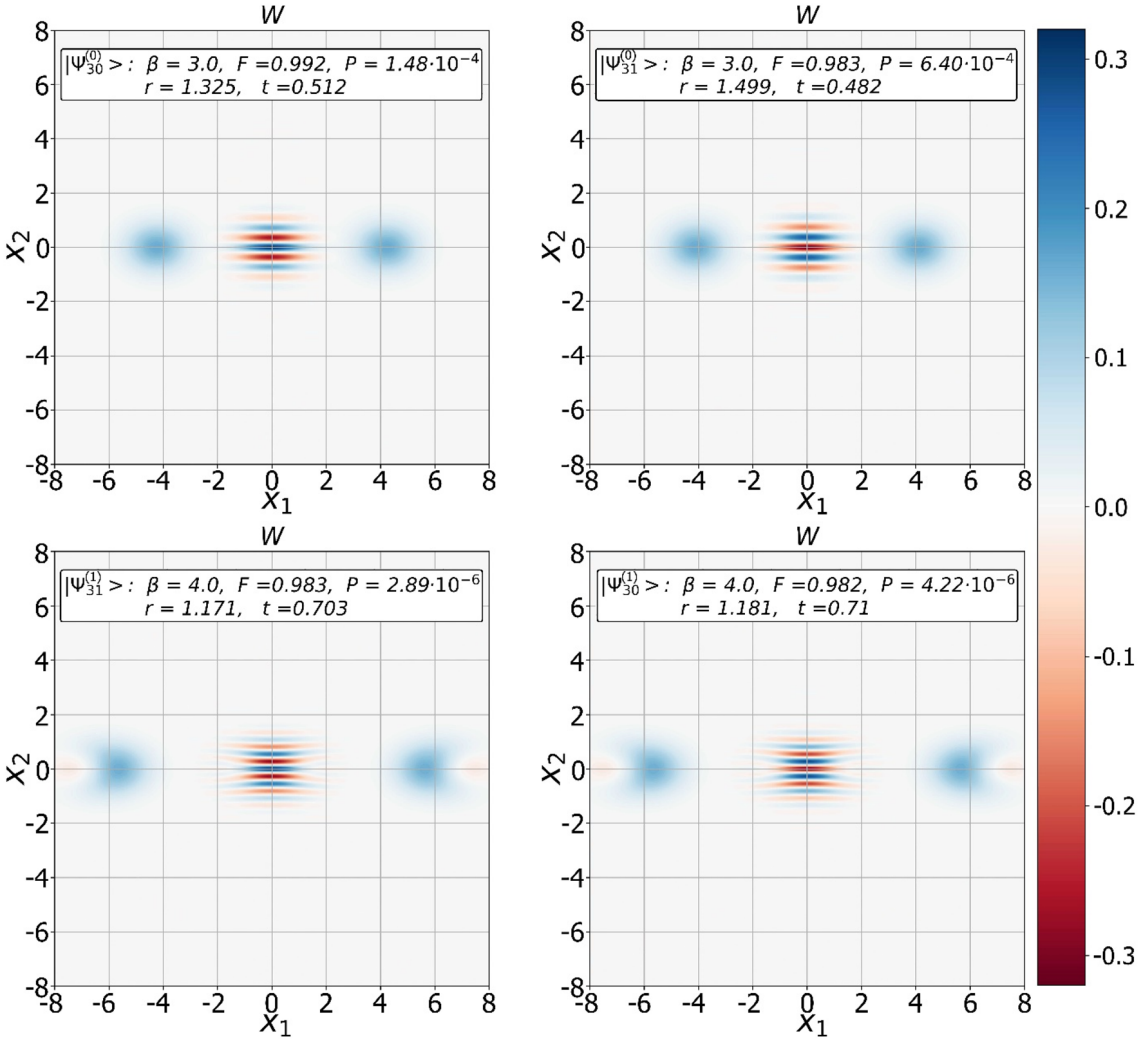
Wigner functions of the even/odd states from ${\Psi }^{\left(0\right)}$ and ${\Psi }^{\left(1\right)}$ families approximating even/odd SCSs with high fidelity.

The property of “some proximity” between SCS-like states and SCSs may suggest that, under certain conditions, the ${\Psi }^{\left(0\right)}$ and ${\Psi }^{\left(1\right)}$ states can be close to even/odd SCSs of a certain amplitude with a fairly high fidelity. Our numerical analysis confirms the conjecture, especially, with $m$ growing. In Fig. 4, we show fidelity between pure measurement-induced states and target SCSs, i.e. ${F}_{2m}^{\left(+\right)}={\left|\langle {SCS}_{+}\left|{\Psi }_{2m}^{\left(0\right)}\right\rangle \right|}^{2}$, ${F}_{2m+1}^{\left(+\right)}={\left|\langle {SCS}_{+}|{\Psi }_{2m+1}^{\left(1\right)}\rangle \right|}^{2}$,

$F_{2m + 1}^{\left(  -  \right)} = {\left| {\left\langle {{SC{S_ - }}}
 \mathrel{\left | {\vphantom {{SC{S_ - }} {{{\Psi }}_{2m + 1}^{\left( 0 \right)}}}}
 \right. \kern-\nulldelimiterspace}
 {{{{\Psi }}_{2m + 1}^{\left( 0 \right)}}} \right\rangle } \right|^2}$, and $${F}_{2m}^{\left(-\right)}={\left|\langle {SCS}_{-}|{\Psi }_{2m}^{\left(1\right)}\rangle \right|}^{2}$$, in dependency on real amplitude $$\beta >0$$ of the SCSs for different values of $$m$$, where SCSs is the superposition of two coherent states $$|\pm \beta \rangle $$

14 $$ \left| {SCS_{ \pm } } \right\rangle = N_{ \pm } \left( \beta \right)\left( {\left| { - \beta } \right\rangle \pm \left| \beta \right\rangle } \right) $$

with $N_{ \pm } \left( \beta \right) = \left( {2\left( {1 \pm exp\left( { - 2\beta^{2} } \right)} \right)} \right)^{ - 1/2}$ being the normalization factors. The graphs indicate on the shaping of both even/odd SCSs of large amplitude with fidelity prevailing $\ge 0.99$ at certain values of $\left( {t,r} \right)$. The maximally high values of the parameters of the output states (amplitude of the SCS, fidelity) are observed in the case of subtraction of either $30$ or $31$ photons. Measurement induced states with fidelity greater than $> 0.99$ are observed for $\beta \le \beta_{0.99}^{\left( 0 \right)} = 3.1$ (top two graphs) and $\beta \le \beta_{0.99}^{\left( 1 \right)} = 4.2$ (bottom two graphs), so that $\beta_{0.99}^{\left( 0 \right)} \le \beta_{0.99}^{\left( 1 \right)}$, where $\beta_{0.99}^{{\left( {0,1} \right)}}$ is amplitude of the even/odd SCS the fidelity of which with generated state exceeds $\ge 0.99$. Thus, the use of states from ${\Psi }^{\left( 1 \right)}$ family allows one to achieve better characteristics (amplitude, fidelity) of the target SCSs compared to use of the states from ${\Psi }^{\left( 0 \right)}$ set with the same value of the parameter $m$. In general, an increase in the number of the subtracted photons leads to a regular gradual increase in the fidelity of the output states. In Fig. 5, we show Wigner functions of the conditioned states $|{\Psi }_{30}^{\left(0\right)}\rangle$ (top left plot), $|{\Psi }_{31}^{\left(0\right)}\rangle$ (top right plot), $|{\Psi }_{31}^{\left(1\right)}\rangle$ (bottom left plot) and $|{\Psi }_{30}^{\left(1\right)}\rangle$ (bottom right picture) the fidelity of which with even/odd SCSs is $\approx 0.99$. The choice of the values $\left(t,r\right)$ is done to provide a sufficiently small value of the squeezing parameter $r$ for its practical implementation.

Maximal values of probability in photon distribution of SMSV state are located near lower number states, while the maximum value in even/odd SCS probability distribution is shifted towards ${n\sim \left|\beta \right|}^{2}$. In the case of the photon subtraction from SMSV, amplitude indexes ${b}_{2n}$ of the original SMSV state receive a displacement by either $m$ $\left({b}_{2n}\to {b}_{2\left(n+m\right)}\right)$ or $m+1$ $\left({b}_{2k}\to {b}_{2\left(k+m+1\right)}\right)$ that chops off more probabilistic terms of the original SMSV distribution. Such index-shifted distribution may become more uniform, especially in the case of a large photon subtraction. But, in addition, the amplitudes of measurement-induced states acquire additional factors which can properly modify the uniform distribution. Choice of appropriate parameters $\left(r,t\right)$ leads to an increase in the amplitudes in the vicinity of the Fock state ${n}_{max}\sim {\left|\beta \right|}^{2}$, leaving them almost unchanged outside the range of Fock states $n<{n}_{max}-{\left|\beta \right|}^{2}$, $n>{n}_{max}+{\left|\beta \right|}^{2}$. It leads to shaping of a new distribution that can largely coincide with target SCSs with fidelity more of $0.99$.

Before, we have considered the possibility of using the states from ${\Psi }^{\left(0\right)}$ and ${\Psi }^{\left(1\right)}$ families as a generator of even/odd SCS in the case of perfect PNR detector with ideal quantum efficiency $\left(\eta =1\right)$. In reality, PNR detectors have imperfect efficiency $\eta <1$, although close to unity^27,28^. To derive the positive-operator values measure (POVM) element of the PNR detector with imperfect detection efficiency $\eta <1$, we model the imperfect detector by placing the fictitious beam splitter of transmissivity $\eta$ before the detector which is responsible for the loss of some of the unregistered photons. So, we have the fidelity of the conditional state in Eq. (3)

15 $$ \begin{gathered} Fid_{2m}^{\left( 0 \right)} = tr\left( {\rho_{2m}^{\left( 0 \right)} \left( {\left| {SCS_{ + } } \right\rangle \left\langle {SCS_{ + } } \right|} \right)} \right)\hfill \\ \quad = \frac{{\mathop \sum \nolimits_{x = 0}^{\infty } \frac{{\left( {1 - \eta } \right)^{2x} }}{{\left( {2x} \right)!}}\left( {\frac{{1 - t^{2} }}{{t^{2} }}} \right)^{2x} y^{{2\left( {m + x} \right)}} Z^{{\left( {2\left( {m + x} \right)} \right)}} \left| {\left\langle {{SCS_{ + } }} \mathrel{\left | {\vphantom {{SCS_{ + } } {{\Psi }_{{2\left( {m + x} \right)}}^{\left( 0 \right)} }}} \right. \kern-\nulldelimiterspace} {{{\Psi }_{{2\left( {m + x} \right)}}^{\left( 0 \right)} }} \right\rangle } \right|^{2} }}{{\mathop \sum \nolimits_{x = 0}^{\infty } \left( {\frac{{\left( {1 - \eta } \right)^{2x} }}{{\left( {2x} \right)!}}\left( {\frac{{1 - t^{2} }}{{t^{2} }}} \right)^{2x} y^{{2\left( {m + x} \right)}} Z^{{\left( {2\left( {m + x} \right)} \right)}} + \frac{{\left( {1 - \eta } \right)^{2x + 1} }}{{\left( {2x + 1} \right)!}}\left( {\frac{{1 - t^{2} }}{{t^{2} }}} \right)^{2x + 1} y^{{2\left( {m + x} \right) + 1}} Z^{{\left( {2\left( {m + x} \right) + 1} \right)}} } \right)}}, \hfill \\ \end{gathered} $$

where $\rho _{2m}^{\left( 0 \right)} = B{S_{12}}\left( {{{\left| {SMSV} \right\rangle }_1}{{\left| 0 \right\rangle }_2}} \right)\left( {{{\left\langle {SMSV} \right|}_1}{{\left\langle 0 \right|}_2}} \right)BS_{12}^ +$ and $tr$ stands for trace over states in mode $1$.

Use of real PNR detector with imperfect quantum efficiency $\eta <1$ reduces the fidelity of the conditional states. Decomposition of the fidelity over parameter $1-\eta$ in Eq. (34) allows one to estimate lower bound of the fidelity in Eq. (35). A sufficiently high fidelity (for example, ${F}_{28}^{\left(0\right)}\left(\eta =0.98\right)=0.979627$ for $\beta =3$, ${F}_{30}^{\left(0\right)}\left(\eta =0.98\right)$ for $\beta =3.2$ comparable with fidelities ${F}_{28}^{\left(0\right)}\left(\eta =1\right)=0.989819$ and ${F}_{30}^{\left(e0\right)}\left(\eta =1\right)=0.988808$ in the case of use of the ideal PNR detector) can be achieved in the case of a high transmission beam splitter $\left(t\to 1\right)$, when contribution of ${g}_{2m,1}^{\left(0\right)}$ in Eq. (34) tends to zero. But the use of high transmission beam splitter leads to decrease by several orders of magnitude in the probability of generating the measurement induced states in Eq. (4) since such BS can redirect an insignificant part of the photons into the measurement mode. Maintaining the success probability in Eq. (4) at an acceptable level does not require the use of high transmission beam splitter. But this can lead to decrease in the lower bound of the fidelity in Eq. (35), since the contribution of the term ${g}_{2m,1}^{\left(0\right)}$ can become significant in Eq. (34), especially, with an increase in the number of extracted photons. Thus, in order to maintain harmony between three parameters (amplitude $\beta$ of the SCSs, fidelity of the measurement induced states with SCSs and success probability) of the even/odd SCSs generator with input SMSV, it is necessary to reduce amount $m$ which defines the number $2m$ of the extracted photons.

Another possible application of the states from ${\Psi }^{\left(0\right)}$ and ${\Psi }^{\left(1\right)}$ families is their use in quantum metrology. Indeed, as can be seen from consideration of generator of large amplitude even/odd SCSs, photon distribution of generated CV states can be shifted towards larger numbers which may increase an average number of photons $\langle n\rangle$. In the case of a possible preservation of the quadrature squeezing property in the generated states (Figs. 2), estimation of phase sensitivity may approach to Heisenberg limit that is proportional to $1/\langle n\rangle$, as in the case of original SMSV states from which they stem. Taking into account the increase in the average number of particles $\langle n\rangle$, the states may become promising for more accurate estimation of the phase sensitivity.

### Deterministic imperfect entangling operation

The generation of two-mode hybrid entangled state can be accomplished by mixing the SMSV with delocalized photon (DP)

16 $$ {\left| {\varphi^{{\left( {DP} \right)}} } \right\rangle}_{23} = a_{0} {\left| {01 } \right\rangle }_{23}+ a_{1} {\left| {10 } \right\rangle}_{23} , $$

occupying simultaneously modes $2$ and $3$, where the amplitudes $a_{0}$ and $a_{1}$ satisfy the normalization condition $\left| {a_{0} } \right|^{2} + \left| {a_{1} } \right|^{2} = 1$. A single photon $\left|1\right\rangle$ can be easily converted to DF by passing it through the beam splitter. In essence, the amplitudes $a_{0}$ and $a_{1}$ are simply the parameters of the below BS in Fig. 1b, namely, its transmittance and reflection coefficients. The mixing of the states on the BS in Eq. (2) (modes $1$ and $2$ are mixed) with the subsequent registration of the measuring outcome in the auxiliary second mode by PNR detector gives rise to the measurement induced hybrid entangled state

17 $${\left| {{\rm{\Delta }}_n^{\left( {DP} \right)}} \right\rangle _{12}} = N_n^{\left( {DP} \right)}\left( {{a_0}{{\left| {{{\Psi }}_n^{\left( 0 \right)}} \right\rangle }_1}{{\left| 1 \right\rangle }_2} + {a_1}B_n^{\left( {DP} \right)}{{\left| {{{\Psi }}_n^{\left( 1 \right)}} \right\rangle }_1}{{\left| 0 \right\rangle }_2}} \right),$$

regardless of the outcome. Here, an integer $n$ can take on values either $n = 2m$ or $n = 2m + 1$. An additional factor $B_{n}^{{\left( {DP} \right)}}$ has the following form: $B_{0}^{{\left( {DP} \right)}} = \fancyscript{r}L_{0}^{\left( 0 \right)} /L_{0}^{\left( 1 \right)} = \fancyscript{r}Z = \sqrt {\left( {1 - t^{2} } \right)/\left( {1 - t^{4} tanh^{2} r} \right)}$, $B_{2m}^{{\left( {DP} \right)}} = 2mL_{2m}^{\left( 0 \right)} /\fancyscript{r}L_{2m}^{\left( 1 \right)} = \left( {2mt^{2} /\sqrt {1 - t^{2} } } \right)\sqrt {G_{2m} /Z^{{\left( {2m} \right)}} }$ and $B_{2m + 1}^{{\left( {DP} \right)}} = \left( {2m + 1} \right)L_{2m + 1}^{\left( 0 \right)} /\fancyscript{r}L_{2m + 1}^{\left( 1 \right)} = \left( {\left( {2m + 1} \right)t^{2} /\sqrt {1 - t^{2} } } \right)\sqrt {G_{2m + 1} /yZ^{{\left( {2m + 1} \right)}} }$. The quantity $N_{n}^{{\left( {DP} \right)}} = \left( {\left| {a_{0} } \right|^{2} + \left| {a_{1} } \right|^{2} \left| {B_{n}^{{\left( {DP} \right)}} } \right|^{2} } \right)^{ - 1/2}$ is the overall normalization factor. Note additional phase transformation $P\left( \pi \right)$ $\left( {P\left( \pi \right)\left| 0 \right\rangle = \left| 0 \right\rangle , P\left( \pi \right)\left| 1 \right\rangle = - \left| 1 \right\rangle } \right)$ in mode of DV state is applied ($P\left(\pi \right)$ is not applied in the case of $n=0$). The success probabilities to conditionally generate the hybrid entangled states in Eq. (17) are the following: $P_{0}^{{\left( {DP} \right)}} = 1/L_{0}^{\left( 0 \right)2} N_{0}^{{\left( {DP} \right)2}}$, $P_{2m}^{{\left( {DP} \right)}} = \left( {1 - t^{2} } \right)^{2m} /\left( {\left( {2m} \right)!L_{2m}^{\left( 0 \right)2} N_{2m}^{{\left( {DP} \right)2}} } \right)$ and $P_{2m + 1}^{{\left( {DP} \right)}} = t^{2} \left( {1 - t^{2} } \right)^{2m + 1} /\left( {\left( {2m + 1} \right)!L_{2m + 1}^{\left( 0 \right)2} N_{2m + 1}^{{\left( {DP} \right)2}} } \right)$.

The micro–macro entanglement in Eq. (17) is formed from ${\Psi }^{\left( 0 \right)}$ and ${\Psi }^{\left( 1 \right)}$ states, therefore, their parities are opposite to each other for any fixed $n$, i.e. $\left\langle {{{\Psi }_{n}^{\left( 1 \right)} }} \mathrel{\left | {\vphantom {{{\Psi }_{n}^{\left( 1 \right)} } {{\Psi }_{n}^{\left( 0 \right)} }}} \right. \kern-\nulldelimiterspace} {{{\Psi }_{n}^{\left( 0 \right)} }} \right\rangle = 0$. Hence, the CV states in mode 1 can be treated as living in a two-dimensional Hilbert space ${\mathcal{H}}_{1}$ with two possible orthogonal basis states $\left\{ {\left| {{\Psi }_{n}^{\left( 0 \right)} } \right\rangle ,\left| {{\Psi }_{n}^{\left( 1 \right)} } \right\rangle } \right\}$. DV qubit lives also in a two-dimensional Hilbert space ${\mathcal{H}}_{2}$ with two apparent orthogonal basis states $\left\{ {\left|0\right\rangle,\left|{1}\right\rangle} \right\}.$ Finally, the state in Eq. (17) can be described in four-dimensional Hilbert space ${\mathcal{H}}_{12}={\mathcal{H}}_{1}\otimes {\mathcal{H}}_{2}$ with four possible orthogonal basis states $\left\{|{\Psi }_{n}^{\left(0\right)}\rangle |0\rangle ,|{\Psi }_{n}^{\left(1\right)}\rangle |0\rangle ,|{\Psi }_{n}^{\left(0\right)}\rangle |1\rangle ,|{\Psi }_{n}^{\left(1\right)}\rangle |1\rangle \right\}$. Using Peres-Horodeski criterion^35^, we can calculate measure of the micro–macro entanglement in Eq. (17)

18 $$ {\mathcal{N}}_{n}^{{\left( {DP} \right)}} { } = \frac{{2\left| {a_{0} } \right|\left| {a_{1} } \right|\left| {B_{n}^{{\left( {DP} \right)}} } \right|}}{{\left| {a_{0} } \right|^{2} + \left| {a_{1} } \right|^{2} \left| {B_{n}^{{\left( {DP} \right)}} } \right|^{2} }}. $$

**Figure 6:**
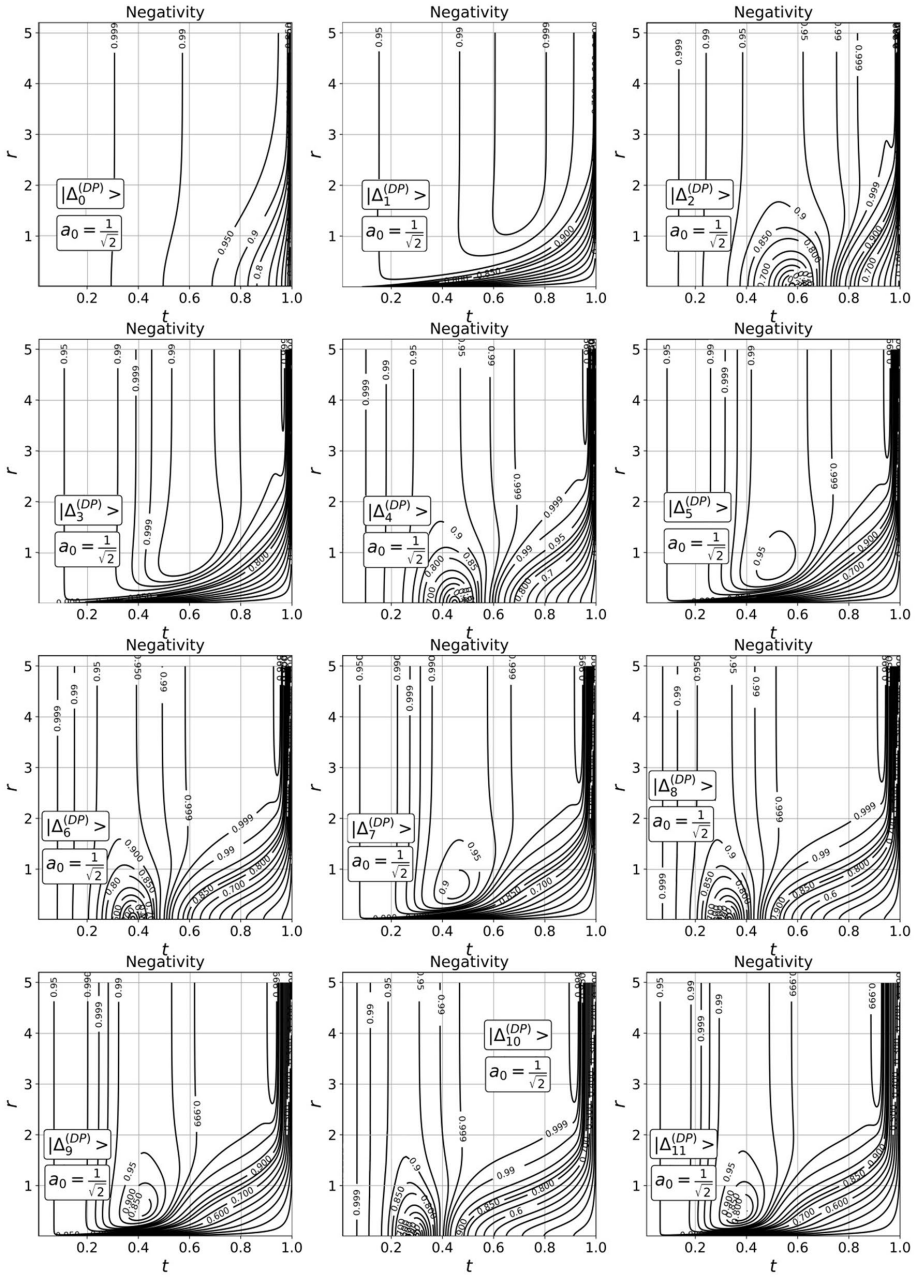
Contour lines of the negativity $\left({\mathcal{N}}_{n}^{\left(DP\right)}=const\right)$ as a function of $t$ and $r$ for the conditional states in Eq. (17) with $n$ varying from $0$ up to $11$ in the case of input balanced delocalized photon in Eq. (16) with ${a}_{0}={a}_{1}=1/\sqrt{2}$.

A distinctive feature of the entangling operation under consideration is the presence of an additional factor ${B}_{n}^{\left(DP\right)}\ne 0$ in output state in Eq. (17) that entails the negativity never takes zero values $\left({0<\mathcal{N}}_{n}^{\left(DP\right)}\le 1\right)$. If ${B}_{n}^{\left(DP\right)}=1$, we can use balanced superposition in Eq. (17), when $\left|{a}_{0}\right|=\left|{a}_{1}\right|=1/\sqrt{2}$ to generate the maximally entangled state with ${\mathcal{N}}_{n}^{\left(DF\right)}=1$. In the case of ${B}_{n}^{\left(DP\right)}\ne 1$, the amplitudes ${a}_{0}$, ${a}_{1}$ of DP can be used to compensate for the contribution of factor ${B}_{n}^{\left(DP\right)}$ $\left(\left|{a}_{0}\right|=\left|{a}_{1}\right|\left|{B}_{n}^{\left(DP\right)}\right|\right)$ to obtain ${\mathcal{N}}_{n}^{\left(DP\right)}=1$ for a certain measuring outcome. Thus, we can talk about deterministic entangling operation which creates micro–macro entanglement in Eq. (17) involving the factor ${B}_{n}^{\left(DP\right)}$ which is an integral feature of the operation. So, in the case $n=0$, the value of ${B}_{0}^{\left(DP\right)}$ does not reach the value $1$ $\left({B}_{0}^{\left(DP\right)}<1\right)$ for any values of the experimental parameters $\left(t,r\right)$. Therefore, in the case, unequal amplitudes $\left|{a}_{0}\right|\ne \left|{a}_{1}\right|$ of DP should be used to compensate for the contribution of ${B}_{0}^{\left(DP\right)}$. In the sense, the entanglement operation is imperfect since it does not guarantee the generation of the maximally entangled state under arbitrary initial conditions. We show in Fig. 6 dependence of the ${\mathcal{N}}_{n}^{\left(DP\right)}$ $\left(n=\mathrm{0,1},2,\dots ,11\right)$ for balanced DF on $r$ and $t$. As can be seen, the range of the values $\left(t,r\right)$ at which the maximum possible negativity of the generated entangled states is observed is significant.

### Realization of hybrid cluster states

Consider an extension of the entanglement operation between CV states of certain parity and photonic states realized on BS. To do it let us make use of TEDP state

19 $$ {\left| {\varphi^{{\left( {TEDP} \right)}} } \right\rangle}_{3456} = a_{0} {\left| {0101 } \right\rangle}_{3456} + a_{1} {\left| {1010 } \right\rangle}_{3456} , $$

which occupy modes $3-6$ with normalization condition ${\left|{a}_{0}\right|}^{2}+{\left|{a}_{1}\right|}^{2}=1$. Note that the entangled state in Eq. (19) can be realized from an initially polarization-entangled state ${|\varphi \rangle }_{34}={a}_{0}{|H\rangle }_{3}{|V\rangle }_{4}+{a}_{1}{|V\rangle }_{3}{|H\rangle }_{4}$ with horizontal $|H\rangle$ and vertical $|V\rangle$ base polarizations by passing it through polarization beam splitters. The procedure of entangling operation involves collective mixing of two SMSV ${|SMSV\rangle }_{1}{|SMSV\rangle }_{2}$ with equal squeezing parameters ${r}_{1}={r}_{2}=r$ (Eq. (1)) with the photonic state (Eq. (19)) on two identical beam splitters ${BS}_{13}$ and ${BS}_{24}$ with arbitrary transmittance and reflectance coefficients with subsequent registration of measurement outcomes in modes $3$ and $4$. This scheme can be directly extended from that in Fig. 1 (right subfigure) by adding an additional BS and PNR detector. The measurement induces hybrid entangled state

20 $${|{\Lambda }_{n,{n}_{1}}^{\left(TEDP\right)}\rangle }_{1234}={N}_{n,{n}_{1}}^{\left(TEDP\right)}\left({a}_{0}{|{\Psi }_{n}^{\left(0\right)}\rangle }_{1}{|{\Psi }_{{n}_{1}}^{\left(1\right)}\rangle }_{2}{|01\rangle }_{34}+{a}_{1}{B}_{n,{n}_{1}}^{\left(TEDP\right)}{|{\Psi }_{n}^{\left(1\right)}\rangle }_{1}{|{\Psi }_{{n}_{1}}^{\left(0\right)}\rangle }_{2}{|10\rangle }_{34}\right),$$

regardless of the measurement outcomes $n$ and ${n}_{1}$, where ${N}_{n,{n}_{1}}^{\left(TEDP\right)}$ is the normalization factor.

The measurement induced state consists of the CV states from either ${\Psi }^{\left(0\right)}$ or ${\Psi }^{\left(1\right)}$ families and components of the delocalized photon. Since the CV states in the same mode belong to different sets, they have opposite parities. The conditional state in Eq. (20) also contains an additional factor ${B}_{n,{n}_{1}}^{\left(TEDP\right)}$ that is integral part of the entanglement operation. But the additional factor is different from the ${B}_{n}^{\left(DP\right)}$ in Eq. (17). So, the additional factors ${B}_{2m,2l}^{\left(TEDP\right)}=\left(m{L}_{2m}^{\left(0\right)}{L}_{2l}^{\left(1\right)}\right)/\left(l{L}_{2l}^{\left(0\right)}{L}_{2m}^{\left(1\right)}\right)$ and ${B}_{2m+\mathrm{1,2}l+1}^{\left(TEDP\right)}=\left(\left(2m+1\right){L}_{2m+1}^{\left(0\right)}{L}_{2l+1}^{\left(1\right)}\right)/\left(\left(2l+1\right){L}_{2l+1}^{\left(0\right)}{L}_{2m+1}^{\left(1\right)}\right)$ become equal to one ${B}_{2m,2m}^{\left(TEDP\right)}={B}_{2m+\mathrm{1,2}m+1}^{\left(TEDP\right)}=1$, when $m=l$. So, in the case of $m=l=0$, the measurement induced state in Eq. (20) becomes three-qubit hybrid cluster state (see section Methods)

21 $$|{HC}_{3}\rangle =\left(|+\rangle |\tilde{0 }\rangle |-\rangle +|-\rangle |\tilde{1 }\rangle |+\rangle \right)/\sqrt{2,}$$

in the case of ${a}_{0}={a}_{1}=1/\sqrt{2}$. The three-qubit HC is composed of the CV states from different families: $|+\rangle =|{\Psi }_{0}^{\left(0\right)}\rangle$ (Eq. (5)), $|-\rangle =|{\Psi }_{0}^{\left(1\right)}\rangle$ (Eq. (8)) and components of delocalized photon: $|\tilde{0 }\rangle =|01\rangle$, $|\tilde{1 }\rangle =|10\rangle$. The CV states $|\pm \rangle$ can be the result of action of Hadamard gate $H$ applied to the CV states of uncertain parity $|{\theta }_{\pm }\rangle =\left(|{\Psi }_{0}^{\left(0\right)}\rangle \pm |{\Psi }_{0}^{\left(1\right)}\rangle \right)/\sqrt{2}$: $H|{\theta }_{+}\rangle =|+\rangle$ and $H|{\theta }_{-}\rangle =|-\rangle$. The success probability to realize the three-qubit HC state is ${P}_{\mathrm{0,0}}^{\left(HL\right)}={Z}^{4}\left({1-t}^{2}\right)/\left({cosh}^{2}r\right)$. In the case of $t\to 1$ and sufficiently small values of the squeezing amplitude $r<1$, the probability ${P}_{\mathrm{0,0}}^{\left(HL\right)}\approx 1/\left({cosh}^{2}r\right)$ $\left(y\approx 0,Z\left(y\right)\approx 1\right)$ prevails over others.

The cluster state in Eq. (21) can be considered as a result of the action of two $control-Z$ (CZ) gates applied to CV states of opposite parities and photonic state $|{HC}_{3}\rangle ={CZ}_{12}{CZ}_{23}\left(|+\rangle |01\rangle |-\rangle +|+\rangle |10\rangle |-\rangle \right)/\sqrt{2}$ by analogy with the formal implementation of the cluster state in Eq. (36). In a real setup, two even SMSV states are used. The parity of the CV state that interacts with the vacuum does not change $\left(|\pm \rangle |0\rangle \to |\pm \rangle \right)$, while the parity of the original SMSV that interacts with a single photon changes to the opposite $\left(|\pm \rangle |1\rangle \to |\mp \rangle \right)$ in the case of vacuum outcomes in auxiliary modes. Taking into account the use of delocalized photon, the relations should be modified $\left(|\pm \rangle |01\rangle \to |\pm \rangle |1\rangle ,|\pm \rangle |10\rangle \to |\mp \rangle |0\rangle \right)$, since one mode of the delocalized photon is used as a measuring. The parity of the initial state of certain parity depends on the parity of the photonic state with which it interacts. It allows us to use the beam splitter as a two-qubit CZ element provided that vacuum outcome is fixed in auxiliary mode.

Consider the possibility of extension of application of the CZ gates on base of the beam splitter to generate a hybrid cluster state of $7$ qubits by conjunction of three-qubit cluster states. To implement such conjunction, we use two HC in Eq. (21) and an additional TEDP state in Eq. (19). As in the case of formal consideration in Eq. (37), one should introduce hybrid states of two qubits: $|{\mathrm{H}}_{1}\rangle =|+\rangle |\tilde{0 }\rangle$, $|{\mathrm{H}}_{2}\rangle =|-\rangle |\tilde{1 }\rangle$, $|{\mathrm{H}}_{3}\rangle =|\tilde{0 }\rangle |-\rangle$ and $|\tilde{1 }\rangle |+\rangle$ to rewrite the three-qubit HC state in Eq. (21) as $|{HC}_{3}\rangle =\left(|{\mathrm{H}}_{1}\rangle |-\rangle +|{\mathrm{H}}_{2}\rangle |+\rangle \right)/\sqrt{2}=\left(|+\rangle |{\mathrm{H}}_{3}\rangle +|-\rangle |{\mathrm{H}}_{4}\rangle \right)/\sqrt{2}$. Two identical beam splitters are used to mix $4$ mode of the first three-qubit HC state with $5$ mode of TEDP and $6$ mode of the second HC state with the 7 mode of the TEDP followed by observing the absence of photons (vacuum states) in the auxiliary measured $5$ and $7$ modes. This operation allows one to connect two three-qubit CH states in Eq. (21) through the TEDP state but the conjunction leads to the appearance of three additional factors like ${B}_{n}^{\left(DP\right)}$ and ${B}_{n,{n}_{1}}^{\left(TEDP\right)}$ (see section Methods).

Let us take advantage of the case when $y$ and, as a consequence ${y}_{1}$, take small values $\left(y,{y}_{1}\ll 1\right)$ which, in particular, can be ensured by using SMSV states with small squeezing amplitude. Then, extra multipliers become ${B}_{\mathrm{0,0}}^{\left(\mathrm{10,01}\right)}={B}_{\mathrm{0,0}}^{\left(\mathrm{01,10}\right)}=t$, ${B}_{\mathrm{0,0}}^{\left(\mathrm{10,11}\right)}={B}_{\mathrm{0,0}}^{\left(\mathrm{11,10}\right)}=\sqrt{2}{t}^{2}$,${B}_{\mathrm{0,0}}^{\left(\mathrm{00,01}\right)}={B}_{\mathrm{0,0}}^{\left(\mathrm{01,00}\right)}=1$ and ${B}_{\mathrm{0,0}}^{\left(\mathrm{00,11}\right)}={B}_{\mathrm{0,0}}^{\left(\mathrm{11,00}\right)}=\sqrt{2}t$ and seven-qubit HC follows from Eq. (38)

22 $$|{HC}_{7}\rangle =\left(\begin{array}{c}\left(|{\mathrm{H}}_{1}\rangle |{-}_{1}\rangle /\sqrt{2}+|{\mathrm{H}}_{2}\rangle |{+}_{1}\rangle \right)|\tilde{0 }\rangle \left(|{-}_{1}\rangle |{\mathrm{H}}_{3}\rangle +|{+}_{2}\rangle |{\mathrm{H}}_{4}\rangle \right)+\\ \left(|{\mathrm{H}}_{1}\rangle |{+}_{2}\rangle +|{\mathrm{H}}_{2}\rangle |{-}_{1}\rangle \right)|\tilde{1 }\rangle \left(|{+}_{1}\rangle |{\mathrm{H}}_{3}\rangle +|{-}_{1}\rangle |{\mathrm{H}}_{4}\rangle /\sqrt{2}\right)\end{array}\right)/\sqrt{6},$$

in the case of use of the balanced BSs with $t=1/\sqrt{2}$. The seven-qubit hybrid cluster state differs from the ideal state in Eq. (37) by the presence of an additional amplitude factor $1/\sqrt{2}$ for two states comprising the HC. In addition to the additional amplitude factor, there is a slight difference in the states $|+\rangle \approx |{+}_{1}\rangle$, $|-\rangle \approx |{-}_{1}\rangle$ but the difference of the states is negligible. The states $|\pm \rangle$ in Eqs. (5,8) depend on the parameter $y$, while the states $|{\pm }_{1}\rangle$ in Eqs. (39,40) are determined by the parameter ${y}_{1}$. More differences exist for the states $|+\rangle$ and $|{+}_{2}\rangle$ in Eq. (41). Using the approach with beam splitters that can play the role of the CZ gate provided that the appropriate outcome in heralding modes, the implementation of large hybrid cluster state composed of more alternating CV states of definite parity and photonic states can be realized by successive conjunction of shorter (by the number of qubits) HC states with help of TEDP state.

## Discussion

We have developed a method of entanglement of continuous CV states of definite parity and photonic states for the purpose of generating more complex entangled states of light. In the model, new families (either ${\Psi }^{\left(0\right)}$ or ${\Psi }^{\left(1\right)}$ in the terminology used) of the CV states of certain parity in dependency on auxiliary photonic states used in the measurement mode is introduced. The states from ${\Psi }^{\left(0\right)}$ family are generated when only SMSV passes through the BS, while the states from ${\Psi }^{\left(1\right)}$ family are produced from the entangled state resulting from the initial mixing of SMSV state with a single photon. Generation of the states from ${\Psi }^{\left(0\right)}$ ${\Psi }^{\left(1\right)}$ families occurs by subtraction of a certain number of photons from original SMSV state. Depending on the number $n$ of detected photons in the measurement mode, the states are finally classified as either $|{\Psi }_{n}^{\left(0\right)}\rangle$ or $|{\Psi }_{n}^{\left(1\right)}\rangle$. The states from ${\Psi }^{\left(0\right)}$ family in Eqs. (3,6) depend only on parameter $0<y<0.5$, while the states from ${\Psi }^{\left(1\right)}$ family in Eqs. (8,10,12) additionally depend on the transmittance coefficient of the beam splitter which can take on arbitrary values in addition to the parameter $y$. Since the extraction of the photons occurs with the loss of information about which Fock state of the initial SMSV the photons are extracted from, the measurement induced states retain the definiteness of its parity (either even or odd). Depending on the number of extracted photons (even, odd), the states from ${\Psi }^{\left(0\right)}$ and ${\Psi }^{\left(1\right)}$ families can be further divided into two subfamilies ($2m-$ and $2m+1-$ heralded) of a certain parity unlike the families themselves, the parity of states of which can be both even and odd.

The potential for using ${\Psi }^{\left(0\right)}$ and ${\Psi }^{\left(1\right)}$ states is quite high. So, the optical scheme in Fig. 1(a) can be used as a generator of both even and odd SCSs. Both states from ${\Psi }^{\left(0\right)}$ family and states from ${\Psi }^{\left(1\right)}$ family can approximate even/odd SCSs of large amplitude with high fidelity, especially, with an increase in the number of extracted photons. In particular, the states from ${\Psi }^{\left(1\right)}$ family can approximate even/odd SCSs of amplitude $\beta =4.2$ with fidelity exceeding $0.99$, when $\mathrm{30,31}$ photons are subtracted from the original SMSV state. The practical implementation of such a generator requires serious progress in PNR detection technologies, namely, the maximum possible increase in its quantum efficiency. The maximum possible quantum efficiency of the PNR detector allows one to maintain the success probability to produce even/odd SCSs at an acceptable level. Since the generation of the even/odd SCSs in the proposed configuration is fragile to the quantum efficiently, the number of subtracted photons is required to reduce or to implement such a generator in a cascade form when the output from one generator is the input to the next one. This deserves a separate consideration. The states from ${\Psi }^{\left(0\right)}$ and ${\Psi }^{\left(1\right)}$ families can also be used in quantum metrology by increasing the average number of photons which can reduce the Heisenberg limit for estimating the phase sensitivity.

We have also shown a possibility of using a beam splitter followed by measurement in one mode to implement an entangled operation for input SMSV and a delocalized photon. The entangling operation realized is deterministic and imperfect. The appearance of an additional integral factor ${B}_{n}^{\left(DP\right)}\ne 1$ determined by the input parameters $\left(t,r\right)$ and also by the measurement outcome can reduce the negativity of the generated hybrid state in Eq. (18). Use of the TEDP state allows one to get rid of the additional factor ${B}_{n,n}^{\left(TEDP\right)}=1$ in the case of identical measurement outcomes. In the interpretation, the beam splitter implements the two-qubit CZ operation for CV states of certain parity and photonic states. The parity of the CV state does not change in the case of its interaction with vacuum on the BS. The interaction of the CV state of definite parity with a single photon changes the parity of the output CV state to the opposite. This makes it possible to produce a three-qubit hybrid state in Eq. (21), for example, in the absence of clicks (vacuum states) in two measured modes. The two-qubit operation CZ based on the beam splitter can be used to connect two three-qubit hybrid states into seven-qubit HC through mixing with additional TEDP on two beam splitters. The output state acquires some additional multiplier in the case of vacuum measurements in two auxiliary modes. Construction of fifteen qubit hybrid cluster state can be implemented using also two beam splitters mixing seven-qubit HC with two modes of the TEDP with subsequent registration, for example, of vacuum outcomes in measured modes. Thus, states from ${\Psi }^{\left(0\right)}$ and ${\Psi }^{\left(1\right)}$ families have the potential to create hybrid cluster states long enough. Further development of the imperfect entangling operation for constructing hybrid cluster states with more number of qubits deserves a separate consideration, for example, in the direction of getting rid of the amplitude factors.

## Methods

### Interaction of *even/odd* Fock states with vacuum and single photon

The basis for derivation of the ${\Psi }^{\left( 0 \right)} -$ (Eqs. (3,6)) and ${\Psi }^{\left( 1 \right)} -$ (Eqs. (8,10,12)) families of CV states is mathematical transformations with creation operators: $a_{1}^{ + } \to ta_{1}^{ + } - \fancyscript{r}a_{2}^{ + }$ and $a_{2}^{ + } \to \fancyscript{r}a_{1}^{ + } + ta_{2}^{ + }$ imposed by the beam splitter in Eq. (2) that distribute initial Fock states to entangled superpostions

23 $$ BS_{12} \left( {\left| {2l} \right\rangle_{1} \left| 0 \right\rangle_{2} } \right) = \mathop \sum \limits_{k = 0}^{2l} \left( { - 1} \right)^{k} t^{2l - k} \fancyscript{r}^{k} \sqrt {\frac{{\left( {2l} \right)!}}{{k!\left( {2l - k} \right)!}}} \left| {2l - k} \right\rangle_{1} \left| k \right\rangle_{2} , $$

24 $$ BS_{12} \left( {\left| {2l + 1} \right\rangle_{1} \left| 0 \right\rangle_{2} } \right) = \mathop \sum \limits_{k = 0}^{2l + 1} \left( { - 1} \right)^{k} t^{2l + 1 - k} \fancyscript{r}^{k} \sqrt {\frac{{\left( {2l + 1} \right)!}}{{k!\left( {2l + 1 - k} \right)!}}} \left| {2l + 1 - k} \right\rangle_{1} \left| k \right\rangle_{2} , $$

25 $$ \begin{gathered} BS_{12} \left( {\left| {2l} \right\rangle_{1} \left| 1 \right\rangle_{2} } \right) = \sqrt {2l + 1} \fancyscript{r}t^{2l} \left| {2l + 1} \right\rangle_{1} \left| 0 \right\rangle_{2} + \hfill \\ \mathop \sum \limits_{k = 0}^{2l} \left( { - 1} \right)^{k} \frac{{t^{2l - k - 1} \fancyscript{r}^{k} }}{k!}\sqrt {\frac{{\left( {2l} \right)!\left( {k + 1} \right)!}}{{\left( {2l - k} \right)!}}} \left( {t^{2} - \frac{2l - k}{{k + 1}}\fancyscript{r}^{2} } \right)\left| {2l - k} \right\rangle_{1} \left| {k + 1} \right\rangle_{2} , \hfill \\ \end{gathered} $$

26 $$ \begin{gathered} BS_{12} \left( {\left| {2l + 1} \right\rangle_{1} \left| 1 \right\rangle_{2} } \right) = \sqrt {2l + 2} \fancyscript{r}t^{2l + 1} \left| {2l + 2} \right\rangle_{1} \left| 0 \right\rangle_{2} + \hfill \\ \mathop \sum \limits_{k = 0}^{2l + 1} \left( { - 1} \right)^{k} \frac{{t^{2l - k} \fancyscript{r}^{k} }}{k!}\sqrt {\frac{{\left( {2l + 1} \right)!\left( {k + 1} \right)!}}{{\left( {2l + 1 - k} \right)!}}} \left( {t^{2} - \frac{2l + 1 - k}{{k + 1}}\fancyscript{r}^{2} } \right)\left| {2l + 1 - k} \right\rangle_{1} \left| {k + 1} \right\rangle_{2} . \hfill \\ \end{gathered} $$

The derivation of the CV states from ${\Psi }^{\left(0\right)}$ and ${\Psi }^{\left(1\right)}$ families follows from the linearity of the BS operator applied to each term of the input SMSV state.

### Derivation of ${\varvec{Z}}\left({\varvec{y}}\right)$

Consider the mathematical inference of the function $Z\left( y \right)$, which is used in derivation of the normalized factors for the states from ${\Psi }^{\left( 0 \right)}$ and ${\Psi }^{\left( 1 \right)}$ sets as well as for their statistical characteristics. To do it let us make use of the $x -$ representation of the squeezed state (SS)${\Psi }_{SS} \left( x \right) = \left\langle {x} \mathrel{\left | {\vphantom {x {SS}}} \right. \kern-\nulldelimiterspace} {SS} \right\rangle$

27 $$ {\Psi }_{SS} \left( x \right) = \frac{\sqrt R }{{\pi^{1/4} }}exp\left( { - \frac{{R^{2} x^{2} }}{2}} \right), $$

where the real parameter $R$ is responsible for state’s “squeezing” properties, as well as $x -$ representation of the coherent state ${\Psi }_{\alpha } \left( x \right) =  \left\langle x{|}\alpha \right\rangle$ with real amplitude $\alpha$

28 $$ {\Psi }_{\alpha } \left( x \right) = \frac{1}{{\pi^{1/4} }}exp\left( { - \frac{{\left( {x - \sqrt 2 \alpha } \right)^{2} }}{2}} \right), $$

where $\left| \alpha \right\rangle = exp\left( { - \left| \alpha \right|^{2} /2} \right)\mathop \sum \limits_{n = 0}^{\infty } \left( {\alpha^{n} /\sqrt {n!} } \right)\left| n \right\rangle$. The inner product of the states in Eqs. (27,28)

29 $$ \left\langle {\alpha } \mathrel{\left | {\vphantom {\alpha {SS}}} \right. \kern-\nulldelimiterspace} {SS} \right\rangle = \mathop \smallint \limits_{ - \infty }^{\infty } {\Psi }_{\alpha } \left( x \right){\Psi }_{SS} \left( x \right)dx = \sqrt {\frac{2R}{{1 + R^{2} }}} exp\left( { - \frac{{R^{2} \alpha^{2} }}{{1 + R^{2} }}} \right) $$

is used to evaluate amplitudes of the SS of even parity $\left|SS\right\rangle = \mathop \sum \limits_{n = 0}^{\infty } a_{2n} \left|2n\right\rangle$ with normalization condition $\mathop \sum \limits_{n = 0}^{\infty } \left| {a_{2n} } \right|^{2} = 1$

30 $$ a_{2n} = \left\langle {2n} \mathrel{\left | {\vphantom {{2n} {SS}}} \right. \kern-\nulldelimiterspace} {SS} \right\rangle = \sqrt {\frac{2R}{{1 + R^{2} }}} \left( {\frac{{1 - R^{2} }}{{2\left( {1 + R^{2} } \right)}}} \right)^{n} \frac{{\sqrt {\left( {2n} \right)!} }}{n!}, $$

when deriving the expression, we used the expansion of the exponent in Eq. (29) in the Taylor series.

Let us introduce the quantity $y = \left( {1 - R^{2} } \right)/\left( {2\left( {1 + R^{2} } \right)} \right)$ to express

31 $$ \frac{{1 + R^{2} }}{2R} = Z\left( y \right) = \frac{1}{{\sqrt {1 - 4y^{2} } }}, $$

as $R^{2} = \left( {1 - 2y} \right)/\left( {1 + 2y} \right)$ that allows one to rewrite the amplitudes of the SS

32 $$ a_{2n} = \frac{{y^{n} \sqrt {\left( {2n} \right)!} }}{\sqrt Z n!}, $$

to get final expression for the function $Z\left( y \right)$

33 $$ \mathop \sum \limits_{n = 0}^{\infty } y^{2n} \frac{{\left( {2n} \right)!}}{{\left( {n!} \right)^{2} }} = Z\left( y \right) = \frac{1}{{\sqrt {1 - 4y^{2} } }}. $$

In particular, if we choose $R = exp\left( { - s} \right)$, then we have $y = tahhs/2$ that gives amplitudes of SMSV: $a_{2n} = b_{2n} /\sqrt {coshr}$ (Eq. (1)). The normalization factors of the $2m,2m + 1 -$ heralded states can be expressed through the derivatives of the expression $Z\left( y \right)$.

### Fidelity of conditional states in the case of $1 - {\varvec{\eta}} \ll 1$

Modern PNR detectors on the base of TES technologies are quite effective^??^ $\left( {\eta \cong 1,\eta < 1} \right)$, which enables considering the fidelity of the states in Eq. (3) in the limit of $1 - \eta \ll 1$

34 $$ Fid_{2m}^{\left( 0 \right)} \left( \eta \right) = Fid_{2m}^{\left( 0 \right)} \left( {\eta = 1} \right)\left( {1 - \left( {1 - \eta } \right)g_{2m,1}^{\left( 0 \right)} + \left( {1 - \eta } \right)^{2} g_{2m,2}^{\left( 0 \right)} + \cdots } \right), $$

where $$g_{2m,1}^{\left( 0 \right)} = t^{2} \left( {1 - t^{2} } \right)L_{2m}^{\left( 0 \right)2} /L_{2m + 1}^{\left( 0 \right)2}$$ and $$g_{2m,2}^{\left( 0 \right)} = {\left( {1 - {t^2}} \right)^2}L_{2m}^{\left( 0 \right)2}/2L_{2\left( {m + 1} \right)}^{\left( 0 \right)2}\left( {2{t^4}L_{2m}^{\left( 0 \right)2}/L_{2m + 1}^{\left( 0 \right)2} + {{{{\left| {\left\langle {{SC{S_ + }}}
 \mathrel{\left | {\vphantom {{SC{S_ + }} {{\rm{\Psi }}_{2\left( {m + 1} \right)}^{\left( 0 \right)}}}}
 \right. \kern-\nulldelimiterspace}
 {{{\rm{\Psi }}_{2\left( {m + 1} \right)}^{\left( 0 \right)}}} \right\rangle } \right|}^2}} \mathord{\left/
 {\vphantom {{{{\left| {\left\langle {{SC{S_ + }}}
 \mathrel{\left | {\vphantom {{SC{S_ + }} {{\rm{\Psi }}_{2\left( {m + 1} \right)}^{\left( 0 \right)}}}}
 \right. \kern-\nulldelimiterspace}
 {{{\rm{\Psi }}_{2\left( {m + 1} \right)}^{\left( 0 \right)}}} \right\rangle } \right|}^2}} {{{\left| {\left\langle {{SC{S_ + }}}
 \mathrel{\left | {\vphantom {{SC{S_ + }} {{\rm{\Psi }}_{2m}^{\left( 0 \right)}}}}
 \right. \kern-\nulldelimiterspace}
 {{{\rm{\Psi }}_{2m}^{\left( 0 \right)}}} \right\rangle } \right|}^2}}}} \right.
 \kern-\nulldelimiterspace} {{{\left| {\left\langle {{SC{S_ + }}}
 \mathrel{\left | {\vphantom {{SC{S_ + }} {{\rm{\Psi }}_{2m}^{\left( 0 \right)}}}}
 \right. \kern-\nulldelimiterspace}
 {{{\rm{\Psi }}_{2m}^{\left( 0 \right)}}} \right\rangle } \right|}^2}}} - 1} \right)$$. Using the definition of $${L}_{2m}^{\left(0\right)}$$ and $${L}_{2m+1}^{\left(0\right)}$$, one can estimate the fidelity in Eq. (34)

35 $$ Fid_{2m}^{\left( 0 \right)} \left( \eta \right) > Fid_{2m}^{\left( 0 \right)} \left( {\eta = 1} \right)\left( {1 - \left( {1 - \eta } \right)t^{2} \left( {1 - t^{2} } \right)tanhr^{2} \left( {n + \left( {m + 1} \right)^{2} } \right)} \right), $$

for $t > 0.4$ to provide $g_{2m,1}^{\left( 0 \right)} \gg g_{2m,2}^{\left( 0 \right)}$ with $n$ the average number of photons of the state $\left| {\Psi_{2m}^{\left( 0 \right)} } \right\rangle .$ The range of values $t < 0.4$ is incompatible with the generation of high-amplitude $\left( {\beta \ge 2} \right)$ even SCS.

### Cluster states

Formally, a cluster state of three qubits can be realized by sequential application of two CZ gates between first and second qubits ${CZ}_{12}$ and second and third qubits ${CZ}_{12}$

36 $$|{C}_{3}\rangle ={CZ}_{12}{CZ}_{23}|+\rangle |+\rangle |-\rangle =\frac{1}{\sqrt{2}}\left(|+\rangle |0\rangle |-\rangle +|-\rangle |1\rangle |+\rangle \right),$$

where $|\pm \rangle =\left(|0\rangle \pm |1\rangle \right)/\sqrt{2}$ and counting qubits goes from left to right. Application of the Hadamard transformation $H$ to the computer basis $|0\rangle$ and $|1\rangle$ results in $H|0\rangle =|+\rangle$ and $H|1\rangle =|-\rangle$.

The cluster states in Eq. (36) can be rewritten in terms of two-qubit states: $|{\Phi }_{1}\rangle =|+\rangle |0\rangle$, $|{\Phi }_{2}\rangle =|-\rangle |1\rangle$, $|{\Phi }_{3}\rangle =|0\rangle |-\rangle$ and $|{\Phi }_{4}\rangle =|1\rangle |+\rangle$ as $|{C}_{3}\rangle =\frac{1}{\sqrt{2}}\left(|{\Phi }_{1}\rangle |-\rangle +|{\Phi }_{2}\rangle |+\rangle \right)=\frac{1}{\sqrt{2}}\left(|+\rangle |{\Phi }_{3}\rangle +|-\rangle |{\Phi }_{4}\rangle \right)$, that enables us to present conjunction of two three-qubit cluster states in Eq. (36) with help of additional qubit $|+\rangle$ located between two three-qubit cluster states in a compact form

37 $$ \begin{gathered} \left| {C_{7} } \right\rangle = CZ_{34} CZ_{45} \left| {C_{3} } \right\rangle \left|+\right\rangle \left| {C_{3} } \right\rangle = \frac{1}{2\sqrt 2 }, \hfill \\ \left( {\begin{array}{*{20}c} {\left( {\left| {{\Phi }_{1} } \right\rangle \left| - \right\rangle + \left| {{\Phi }_{2} } \right\rangle \left| + \right\rangle } \right)\left|0\right\rangle\left( {\left| +\right\rangle \left|{\Phi }_{3}\right\rangle + \left| - \right\rangle \left|{\Phi }_{4}\right\rangle } \right) + } \\ {\left( {\left| {{\Phi }_{1} } \right\rangle \left| + \right\rangle \left| {{\Phi }_{2} } \right\rangle \left| - \right\rangle } \right)\left| 1 \right\rangle \left( {\left| - \right\rangle \left| {{\Phi }_{3} } \right\rangle + \left| + \right\rangle \left| {{\Phi }_{4} } \right\rangle } \right)} \\ \end{array} } \right). \hfill \\ \end{gathered} $$

### Conjunction of the hybrid cluster states

Collective mixing of two three-qubit HC states in Eq. (21) with TEDP in Eq. (19) on two identical BSs $BS_{67} BS_{45} \left( {\left| {HC_{3} } \right\rangle_{1234} {\left| {\varphi^{{\left( {TEDP} \right)}} } \right\rangle}_{5789} \left| {HC_{3} } \right\rangle_{6,10,11,12} } \right)$ leads to the generation of the following hybrid entangled state

38 $$ \left| {{\Lambda }_{0,0}^{\left( 7 \right)} } \right\rangle = N_{0,0}^{\left( 7 \right)} \left( \begin{gathered} B_{0,0}^{{\left( {10,01} \right)}} \left| {{\text{H}}_{1} } \right\rangle \left| { -_{1} } \right\rangle \left| {01} \right\rangle \left| { -_{1} } \right\rangle \left|{\text{H}}_{3} \right\rangle + B_{0,0}^{{\left( {10,11} \right)}} \left| {{\text{H}}_{1} } \right\rangle \left| { -_{1} } \right\rangle \left| {01} \right\rangle \left| { +_{2} } \right\rangle \left| {{\text{H}}_{4} } \right\rangle + \hfill \\ B_{0,0}^{{\left( {00,01} \right)}} \left| {{\text{H}}_{2} } \right\rangle \left| { +_{1} } \right\rangle \left| {01} \right\rangle \left| { -_{1} } \right\rangle \left|{\text{H}}_{3} \right\rangle + B_{0,0}^{{\left( {00,11} \right)}} |{\text{H}}_{2} \left| { +_{1} } \right\rangle \left| {01} \right\rangle \left| { +_{2} } \right\rangle \left| {{\text{H}}_{4} } \right\rangle + \hfill \\ B_{0,0}^{{\left( {11,00} \right)}} \left| {{\text{H}}_{1} } \right\rangle \left| { +_{2} } \right\rangle \left| {10} \right\rangle \left| { +_{1} } \right\rangle \left|{\text{H}}_{3} \right\rangle + B_{0,0}^{{\left( {11,10} \right)}} \left| {{\text{H}}_{1} } \right\rangle \left| { +_{2} } \right\rangle \left| {10} \right\rangle \left| { -_{1} } \right\rangle \left| {{\text{H}}_{4} } \right\rangle + \hfill \\ B_{0,0}^{{\left( {01,00} \right)}} |{\text{H}}_{2} \left| { -_{1} } \right\rangle \left| {10} \right\rangle \left| { +_{1} } \right\rangle \left| {{\text{H}}_{3} } \right\rangle + B_{0,0}^{{\left( {01,10} \right)}} \left| {{\text{H}}_{2} } \right\rangle \left| { -_{1} } \right\rangle \left| {10} \right\rangle \left| { -_{1} } \right\rangle \left| {{\text{H}}_{4} } \right\rangle \hfill \\ \end{gathered} \right), $$

provided that nothing (vacuum) is registered in the $5$ th and $7$ th auxiliary modes. Here, the additional factors are the following: $B_{0,0}^{{\left( {10,01} \right)}} = B_{0,0}^{{\left( {01,10} \right)}} = tZ\left( {y_{1} } \right)/Z\left( y \right)$, $B_{0,0}^{{\left( {10,11} \right)}} = B_{0,0}^{{\left( {11,10} \right)}} = \sqrt 2 t^{2} Z^{2} \left( {y_{1} } \right)\sqrt {1 + 2y_{1}^{2} } /Z^{2} \left( y \right)$,$B_{0,0}^{{\left( {00,01} \right)}} = B_{0,0}^{{\left( {01,00} \right)}} = 1$ and $B_{0,0}^{{\left( {00,11} \right)}} = B_{0,0}^{{\left( {11,00} \right)}} = \sqrt 2 tZ\left( {y_{1} } \right)\sqrt {1 + 2y_{1}^{2} } /Z\left( y \right)$ and $N_{0,0}^{\left( 7 \right)}$ is the overall normalization factor. Note that the CV states $\left| { \pm_{1} } \right\rangle$ and $\left| { +_{2} } \right\rangle$ of definite parity differ from $\left| + \right\rangle$ in Eq. (5) and $\left| - \right\rangle$ in Eq. (8)

39 $$ \left| { +_{1} } \right\rangle = \frac{1}{{\sqrt {Z\left( {y_{1} } \right)} }}\mathop \sum \limits_{n = 0}^{\infty } y_{1}^{n} \frac{{\sqrt {\left( {2n} \right)!} }}{n!}\left| {2n} \right\rangle , $$

40 $$ \left| { -_{1} } \right\rangle = \frac{1}{{Z\left( {y_{1} } \right)^{3/2} }}\mathop \sum \limits_{n = 0}^{\infty } y_{1}^{n} \frac{{\sqrt {\left( {2n} \right)!} }}{n!}\sqrt {2n + 1} \left| {2n + 1} \right\rangle , $$

41 $$ \left| { +_{2} } \right\rangle = \frac{1}{{\sqrt {2Z^{5} \left( {y_{1} } \right)\left( {1 + 2y_{1}^{2} } \right)} }}\mathop \sum \limits_{n = 0}^{\infty } y_{1}^{n} \frac{{\sqrt {\left( {2n} \right)!} }}{n!}\sqrt {\left( {2n + 1} \right)\left( {2n + 2} \right)} \left| {2n + 2} \right\rangle , $$

where $y_{1} = yt^{2}$. The success probability to generate the seven-qubit HC state is $P_{0,0}^{\left( 7 \right)} = \left( {1 - t^{2} } \right)Z^{4} \left( {y_{1} } \right)/Z^{2} \left( y \right)N_{0,0}^{\left( 7 \right)2}$.
